# Supplementary material for: Versatile strategy for homogeneous drying patterns of dispersed particles
Source: Nat Commun. 2022 May 23;13:2840. doi: 10.1038/s41467-022-30497-z (PMC9126951; doi:10.1038/s41467-022-30497-z)
Supplement: Supplementary file 1 — Supplementary Information [file 41467_2022_30497_MOESM1_ESM.pdf]

Versatile strategy for homogeneous drying patterns of dispersed particles

Supplementary Information

Marcel Rey[a,b,c], Johannes Walter[a,b], Johannes Harrer[a,b], Carmen Morcillo Perez[c], Salvatore Chiera[a,b], Sharanya Nair[a,b], Maret Ickler[a,b], Alesa Fuchs[a,b], Mark Michaud[a,b], Maximilian J. Uttinger[a,b], Andrew B. Schofield[c], Job H. J. Thijssen[c], Monica Distaso[a,b], Wolfgang Peukert[a,b], Nicolas Vogel[a,b]

[a] Institute of Particle Technology (LFG), Friedrich-Alexander-Universität Erlangen-Nürnberg (FAU), Cauerstrasse 4, 91058 Erlangen, Germany
[b] Interdisciplinary Center for Functional Particle Systems (FPS), Friedrich-Alexander-Universität Erlangen-Nürnberg (FAU), Haberstrasse 9a, 91058 Erlangen, Germany
[c] School of Physics & Astronomy, The University of Edinburgh, Peter Guthrie Tait Road, Edinburgh, EH9 3FD, UK.

Table of Contents

Supplementary Figures: 2
Supplementary Table: Experimental details 24
Supplementary Discussion 1: Droplet drying behavior 28
Supplementary Discussion 2: Flow analysis within drying droplets: 29
Supplementary Discussion 3: Homogeneity of the deposit and effect of particle type and shape 32
Supplementary Discussion 4: Concentration calculations to obtain a particle monolayer: 33
Supplementary Discussion 5: Exception: Sterically-stabilized particles 34
Supplementary Note 1: Guide for experimentalists 35
Supplementary References 36

Supplementary Figures:

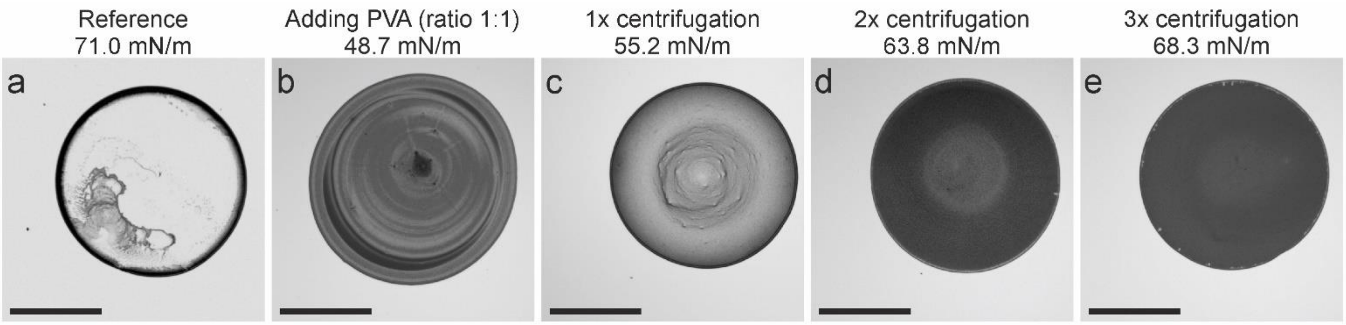

Supplementary Figure 1: Prevention of the coffee ring effect of a PS particle dispersion by adding PVA and subsequent removal of free PVA by centrifugation and redispersion in water. a) Reference particle dispersion in pure water. b-e) Addition of PVA (PS/PVA wt-% ratio: 1:1) without centrifugation and redispersion (b), and with once (c), twice (d) and thrice (e) centrifugation and redispersion. Scale bar: 1 mm.

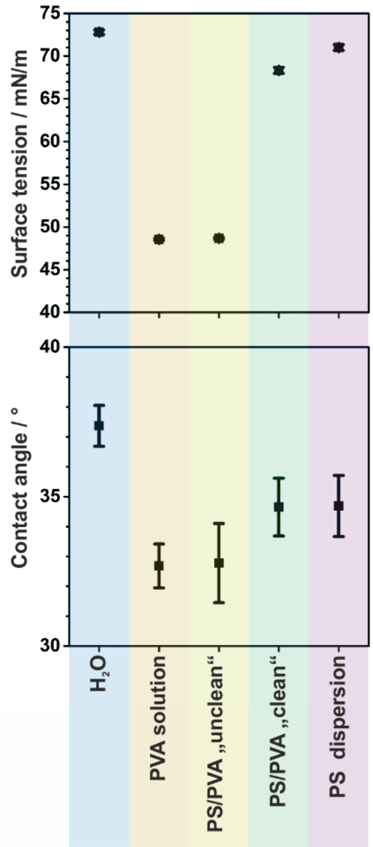

Supplementary Figure 2: Surface tension (top) and contact angle (bottom) measured for the respective dispersions including the standard deviation as error bar. Free PVA causes a decrease in surface tension. After three times cleaning by centrifugation and redispersion against water, the “clean” dispersion displays a similar surface tension and contact angle as the pure PS dispersion.

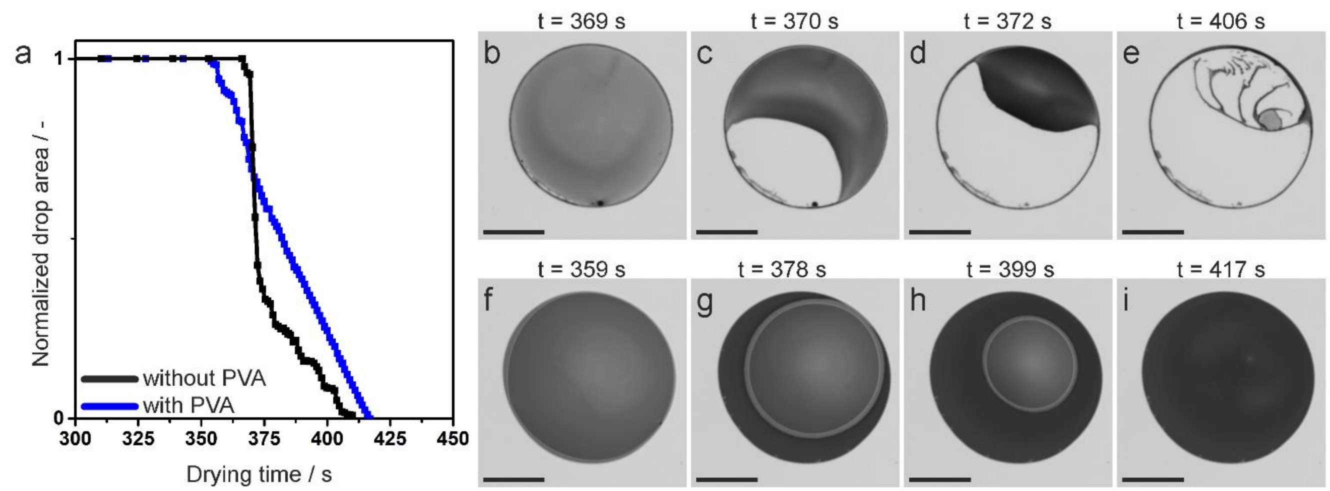

Supplementary Figure 3: Drying dynamics of droplets of spherical PS particle dispersions prepared by embedding in PVA matrix and subsequent purification by centrifugation and redispersion. a) Normalized drop area as a function of drying time. b-i) Snapshots during the drying with (b-e) and without (f-i) PVA removal. b-e) The non-adsorbed and adsorbed PVA was removed by centrifugation and redispersion in an IPA/water mixture. The images correspond to the main text Figure 2e and Supplementary Movie 5. f-i) Only non-adsorbed PVA was removed by centrifugation and redispersion in water. The images correspond to the main text Figure 2b and Supplementary Movie 3. When the adsorbed PVA is removed, the droplet rapidly depins (black line), whereas there is a continuous decrease in droplet area for the particles with adsorbed PVA (blue line). Scale bar: 1 mm.

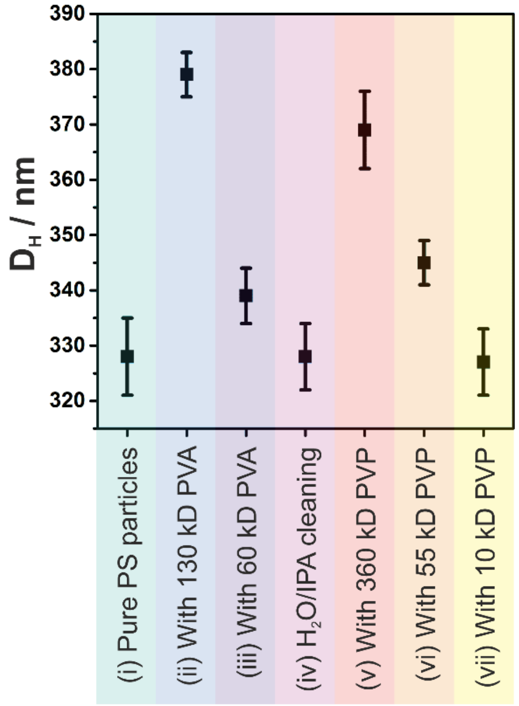

Supplementary Figure 4: Hydrodynamic diameter (D_H) and standard deviation of the respective particle dispersions measured by dynamic light scattering. (i) Pure PS particles. After mixing the PS particles with 130 kDa PVA (ii) or 60 kDa PVA (iii) and subsequent removal of non-adsorbed PVA by centrifugation and redispersion in water, we observe an increase in the hydrodynamic diameter, indicative of a polymeric shell of the physisorbed PVA. We observe a decrease in D_H with decreasing PVA chain length. (iv) Additionally washing the particles with 130 kDa PVA (ii) with H_2O/IPA mixture removes the physisorbed PVA, as indicated by the decrease in hydrodynamic radius to the value of the pure PS dispersion. (v-vii) PS particles were mixed with 360 kDa PVP (v), 55 kDa PVP (vi) and 10 kDa PVP (vii) and excess PVP was removed by centrifugation and redispersion in water. Similar to the case of PVA, the hydrodynamic radius increases by the addition of PVP, indicating the formation of a physisorbed polymer shell. We further notice a decrease in D_H with decreasing PVP chain length.

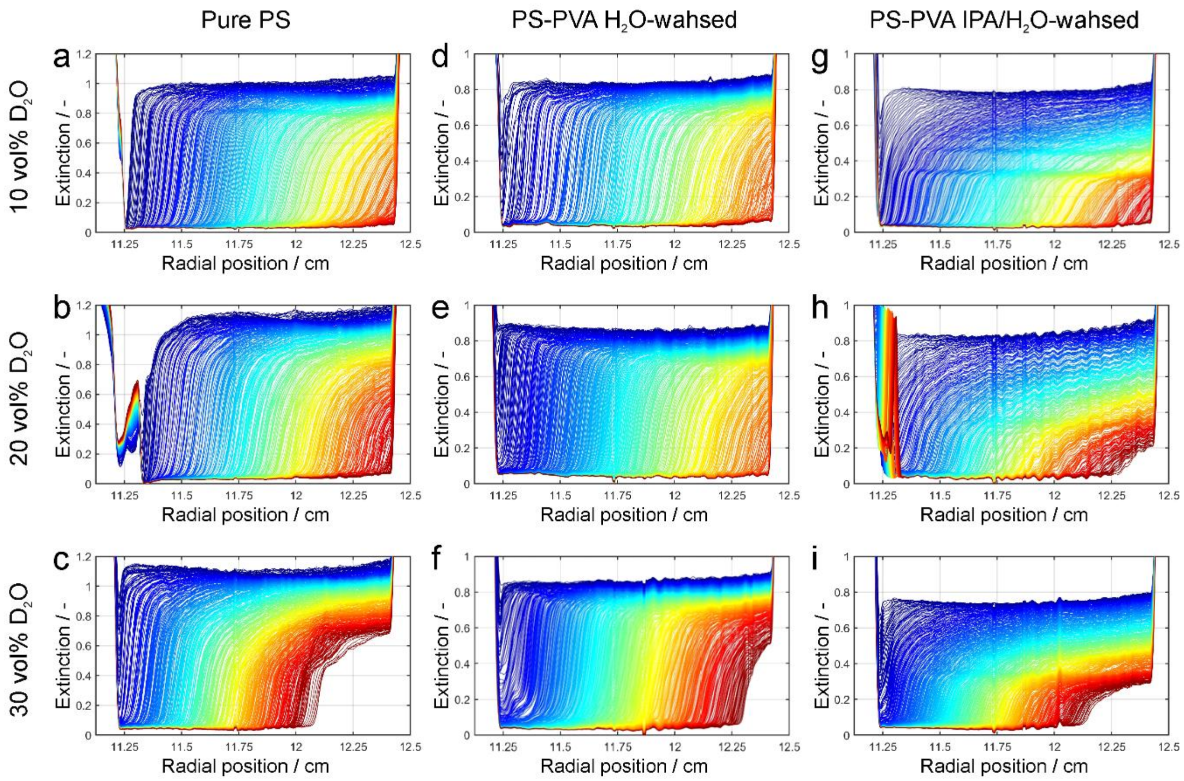

Supplementary Figure 5: Sedimentation profiles for (a-c) pure PS particles, (d-f) H_2O-washed PS-PVA particles and (g-i) IPA/H_2O-washed PS-PVA particles measured in (a,d,g) 10 vol-%, (b,e,h) 20 vol-% and (c,f,i) 30 vol-% D_2O in H_2O. Blue to red color indicates increasing experimental time for the measured sedimentation profiles.

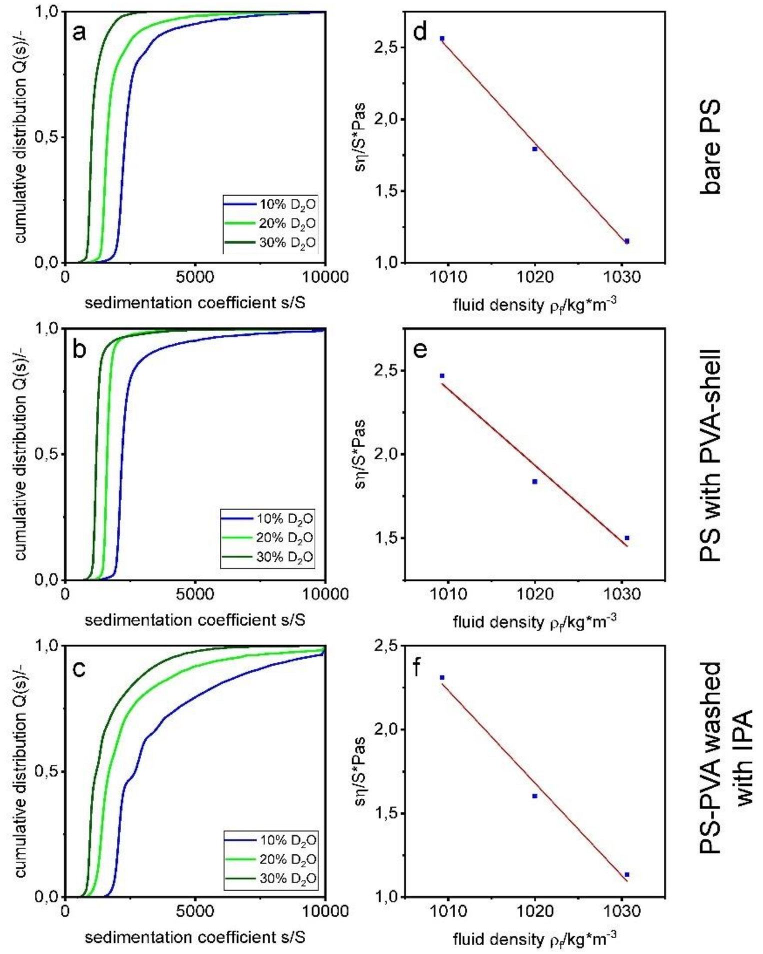

Supplementary Figure 6: Apparent sedimentation coefficient distributions and determination of the particle density. a-c) Cumulative sedimentation coefficient distributions for the three different particle systems. d-f) Product of the sedimentation coefficient and viscosity s*η versus solution density plots. The anhydrous density is the density at which s*η equals zero, thus it is the density of the medium at which the particle is neutrally buoyant and neither sediments nor floats.

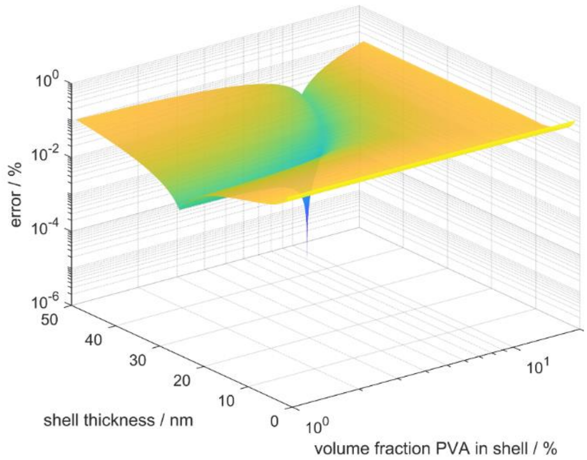

Supplementary Figure 7: Combined error of sedimentation coefficient and anhydrous density calculated for different combinations of PVA volume fraction and shell thickness (see Method section for relevant equations) when compared to the input values derived from the experiment. A well-defined minimum is observable which allows identification of the true PVA volume fraction (4.86 %) and shell thickness (30.1 nm).

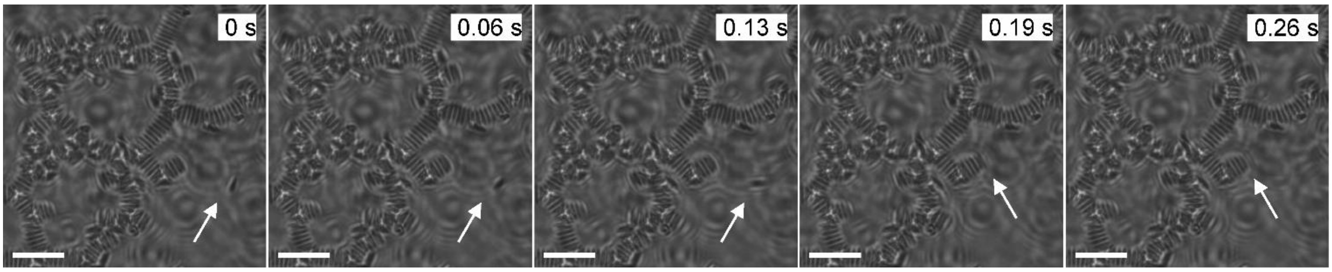

Supplementary Figure 8: Optical microscopy image of an ellipsoidal particle with PVA shell breaching the air/water interface, which then goes on to accumulate at a neighboring particle aggregate due to attractive quadrupolar capillary interactions. The image series are zoomed-in snapshots of Supplementary Movie 10. Scale bar: 10 µm.

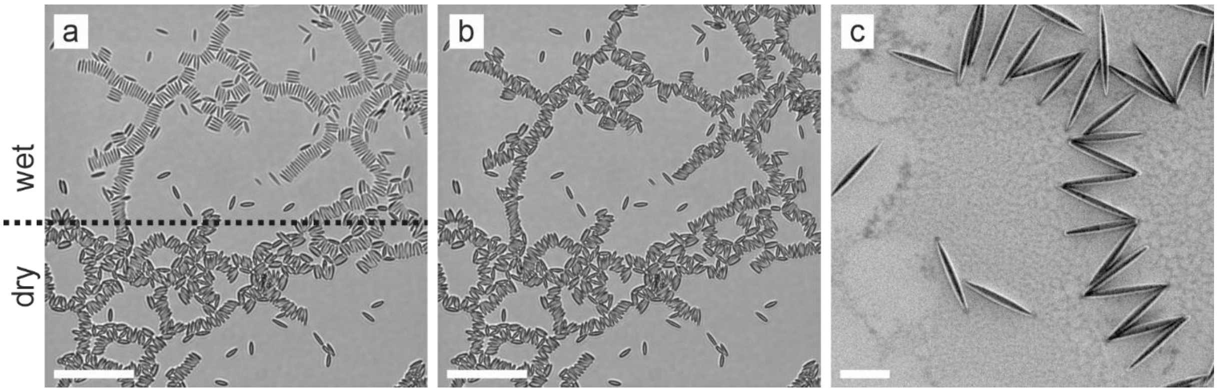

Supplementary Figure 9: Particle movement of ellipsoids with PVA shell during drying. a,b) Optical microscopy images of ellipsoids with PVA shell during the last stage of drying. a) Partially dried. b) Fully dried. The particles rotate towards each other under the effect of capillary forces. Scale bar: 20 µm. c) SEM images of smaller ellipsoidal particles after drying assembled in a zig-zag pattern originating from the non-close packing at the liquid interface and in-plane rotation during the last stage of drying. Scale bar: 1 µm.

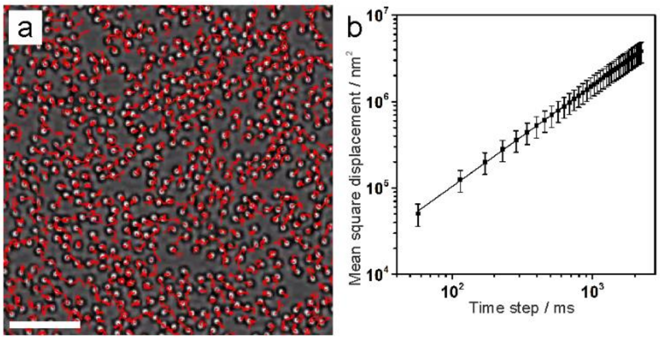

Supplementary Figure 10: Particle tracking of spherical particles with PVA shell confined at the air/water interface from optical microscopy images in the center of an evaporating droplet. a) Particle tracking reveals the diffusion of each particle. b) Mean square displacement and standard deviation vs time step. Scale bar: 10 µm.

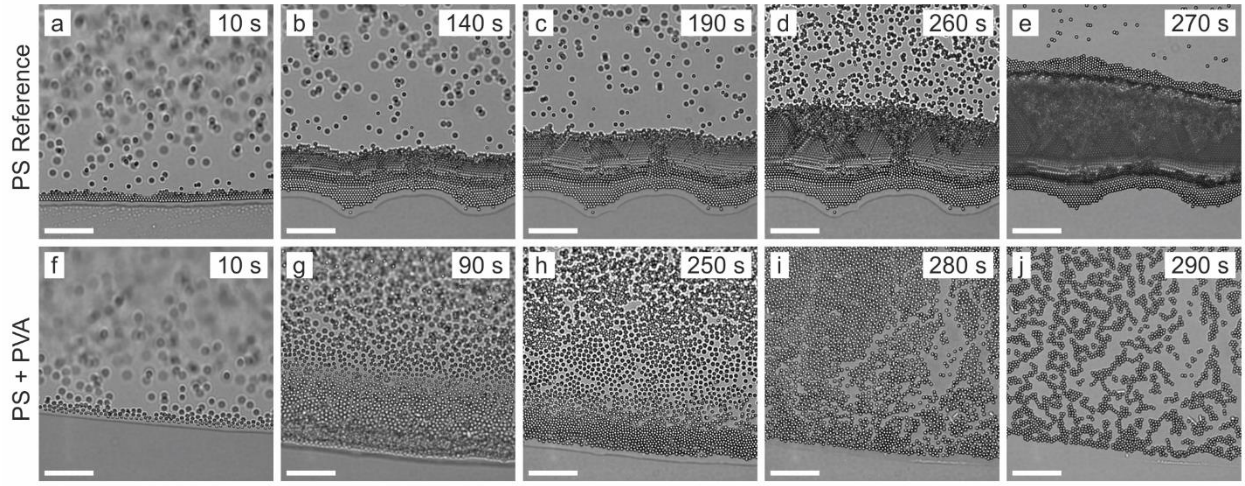

Supplementary Figure 11: High magnification microscopy images during drying of colloidal dispersions. a-e) In dispersions of pure PS colloids, the particles accumulate at the drop edge and form a three-dimensional layer. f-j) PS particles coated with PVA are repelled by the PVA shell and are not in close contact (f). They preferentially adsorb to the air/water interface (g) in a non-close packed arrangement, leading to a uniform drying behavior. Scale bar: 20 µm.

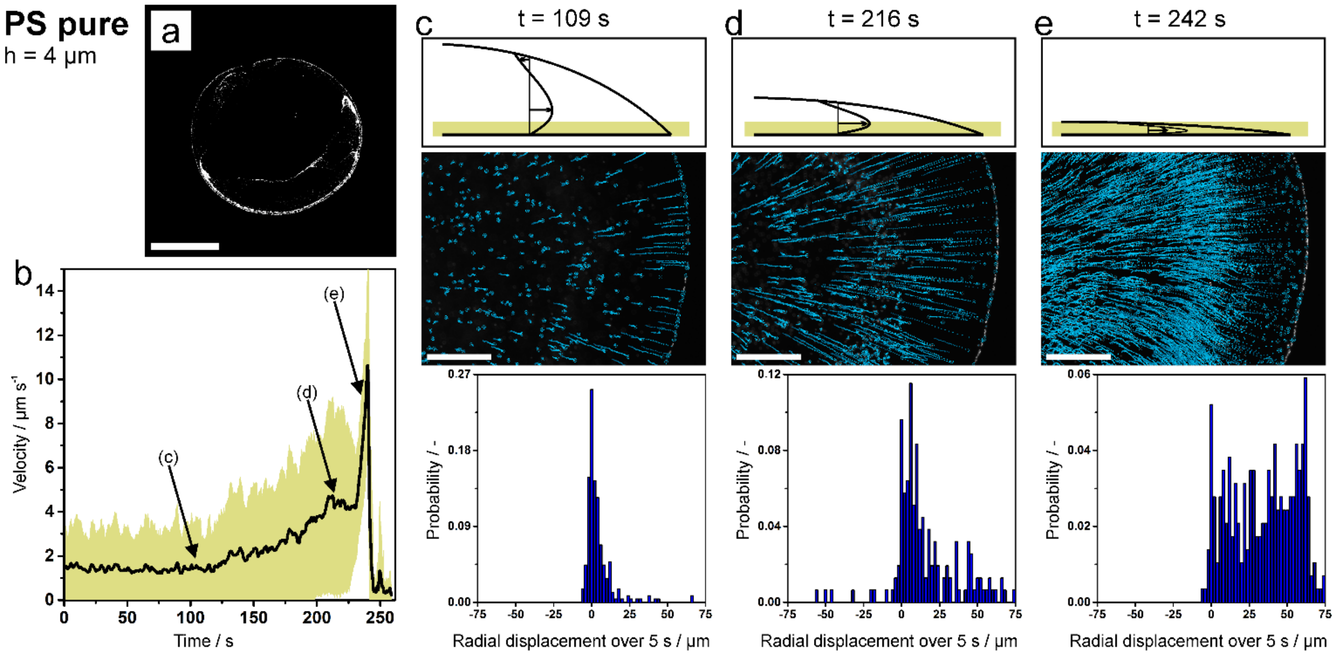

Supplementary Figure 12: Flow analysis within a pure PS particle dispersion droplet containing fluorescent tracer particles measured by fluorescence microscopy with the focal plane set 4 µm above the substrate. a) Fluorescent microscopy image of the deposit after drying. Scale bar: 1 mm. b) Average particle velocity measured over the duration of drying, with the standard deviation shown in light green. c-e) Snapshots of the particle flow for different times: Top: Schematic illustration of the focal plane position relative to the droplet shape and the direction of the flow within the droplet. Middle: Snapshots of the particle trajectory. The open circle corresponds to the tracked position of the particle and the dotted line shows the trajectory of the particles over the last 25 s. The snapshots correspond to Supplementary Movie 12. Scale Bar: 200 µm. Bottom: Histogram of the radial particle displacement over 5 s. Negative values correspond to an inward flow and positive values to an outward flow.

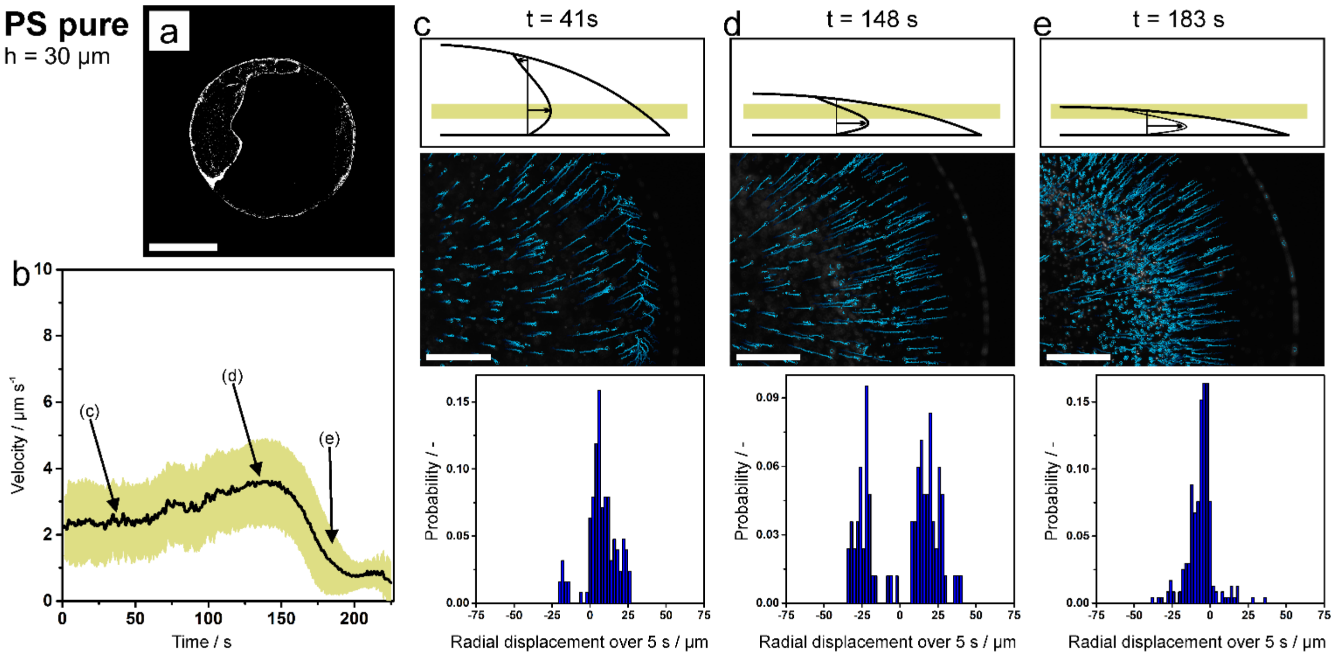

Supplementary Figure 13: Flow analysis within a pure PS particle dispersion droplet containing fluorescent tracer particles measured by fluorescence microscopy with the focal plane set 30 µm above the substrate. a) Fluorescent microscopy image of the deposit after drying. Scale bar: 1 mm. b) Average particle velocity measured over the duration of drying, with the standard deviation shown in light green c-e) Snapshots of the particle flow for different times: Top: Schematic illustration of the focal plane position relative to the droplet shape and the direction of the flow within the droplet. Middle: Snapshots of the particle trajectory. The open circle corresponds to the tracked position of the particle and the dotted line shows the trajectory of the particles over the last 25 s. The snapshots correspond to Supplementary Movie 13. Scale Bar: 200 µm. Bottom: Histogram of the radial particle displacement over 5 s. Negative values correspond to an inward flow and positive values to an outward flow.

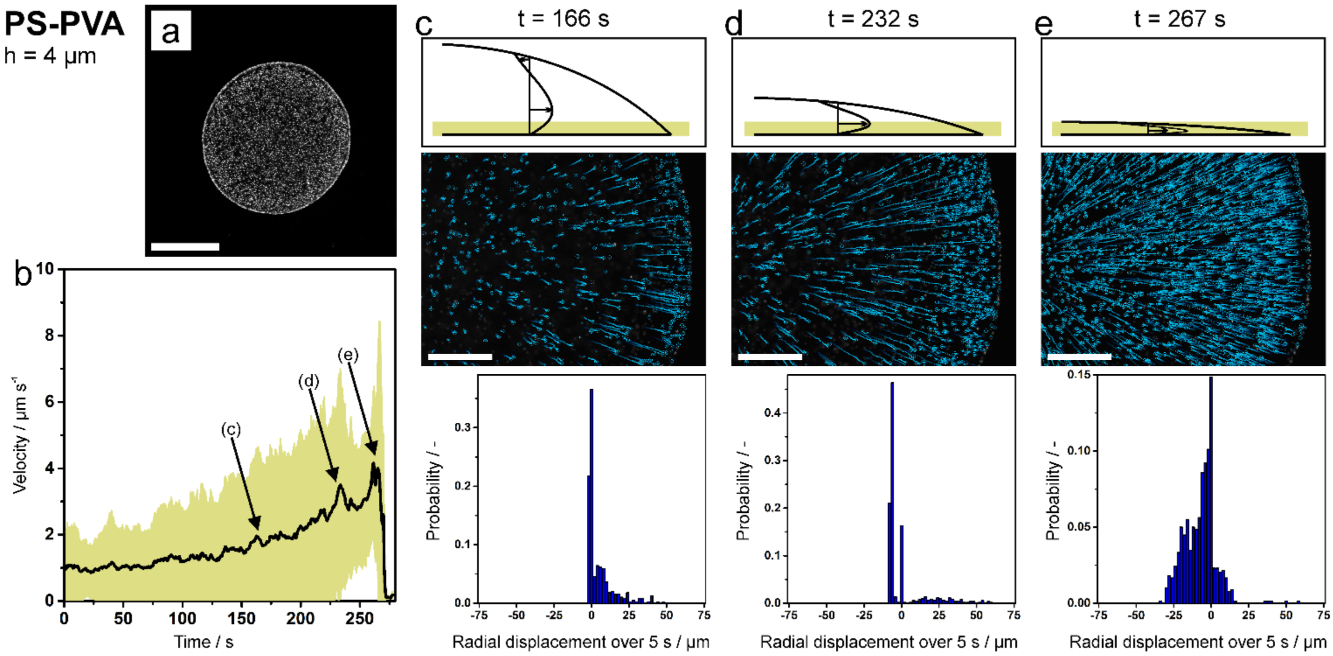

Supplementary Figure 14: Flow analysis within a PVA-modified PS particle suspension droplet containing fluorescent tracer particles measured by fluorescence microscopy with the focal plane set 4 µm above the substrate. a) Fluorescent microscopy image of the deposit after drying. Scale bar: 1 mm. b) Average particle velocity measured over the duration of drying, with the standard deviation shown in light green c-e) Snapshots of the particle flow for different times: Top: Schematic illustration of the focal plane position relative to the droplet shape and the direction of the flow within the droplet. Middle: Snapshots of the particle trajectory. The open circle corresponds to the tracked position of the particle and the dotted line shows the trajectory of the particles over the last 25 s. The snapshots correspond to Supplementary Movie 14. Scale Bar: 200 µm. Bottom: Histogram of the radial particle displacement over 5 s. Negative values correspond to an inward flow and positive values to an outward flow.

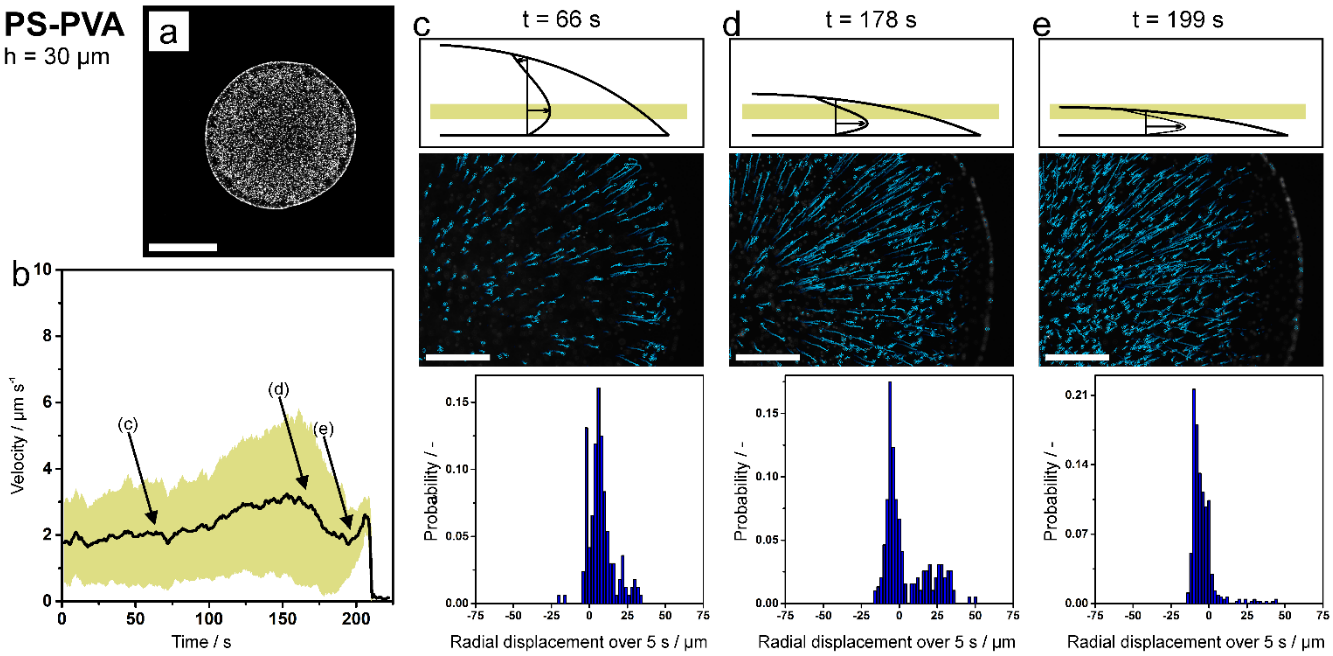

Supplementary Figure 15: Flow analysis within a PVA-modified PS particle suspension droplet containing fluorescent tracer particles measured by fluorescence microscopy with the focal plane set 30 µm above the substrate. a) Fluorescent microscopy image of the deposit after drying. Scale bar: 1 mm. b) Average particle velocity measured over the duration of drying, with the standard deviation shown in light green c-e) Snapshots of the particle flow for different times: Top: Schematic illustration of the focal plane position relative to the droplet shape and the direction of the flow within the droplet. Middle: Snapshots of the particle trajectory. The open circle corresponds to the position of the tracked particle and the dotted line shows the trajectory of the particles over the last 25 s. The snapshots correspond to Supplementary Movie 15. Scale Bar: 200 µm. Bottom: Histogram of the radial particle displacement over 5 s. Negative values correspond to an inward flow and positive values to an outward flow.

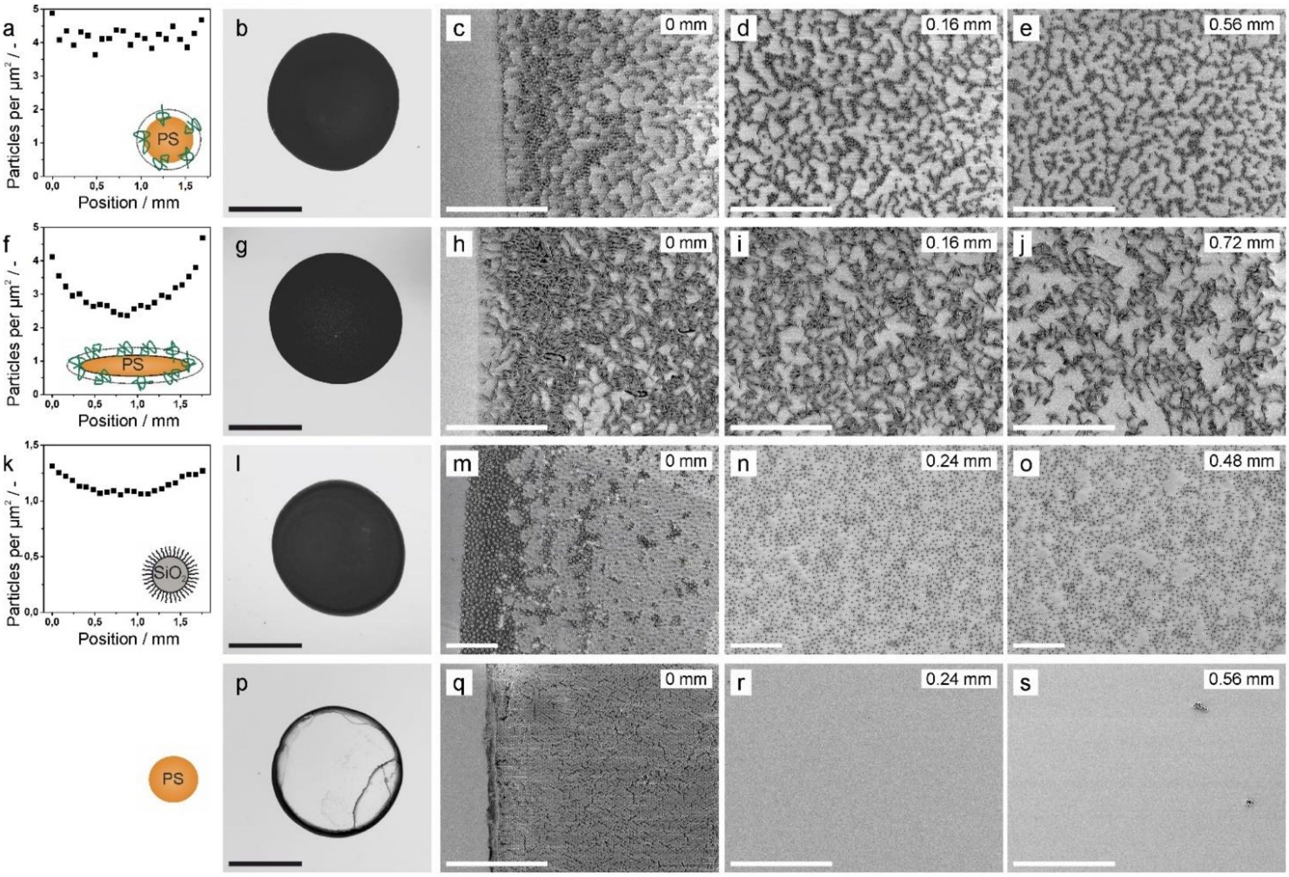

Supplementary Figure 16: Quantitative scanning electron microscopy (SEM) analysis of the particle distribution through the dried deposits. a-e) Spherical PS particles with physisorbed PVA. f-j) Ellipsoidal PS particles with physisorbed PVA. k-o) SiO_2-graft-PNIPAM particles. p-s) Pure spherical PS particles. a,f,k) Particles per area distribution through the dried deposit. SEM images were taken each 80 µm and analyzed using the ImageJ tool "cell counter". Note that the pure reference sample (bottom row) could not be quantified in this way due to the large density at the coffee ring. b,g,l,p) Optical microscopy images of the dried deposits. Scale bar: 1 mm. c-e,h-j,m-o,q-s) Representative SEM images at different positions (inset) from the contact line. Scale bar: 10 µm.

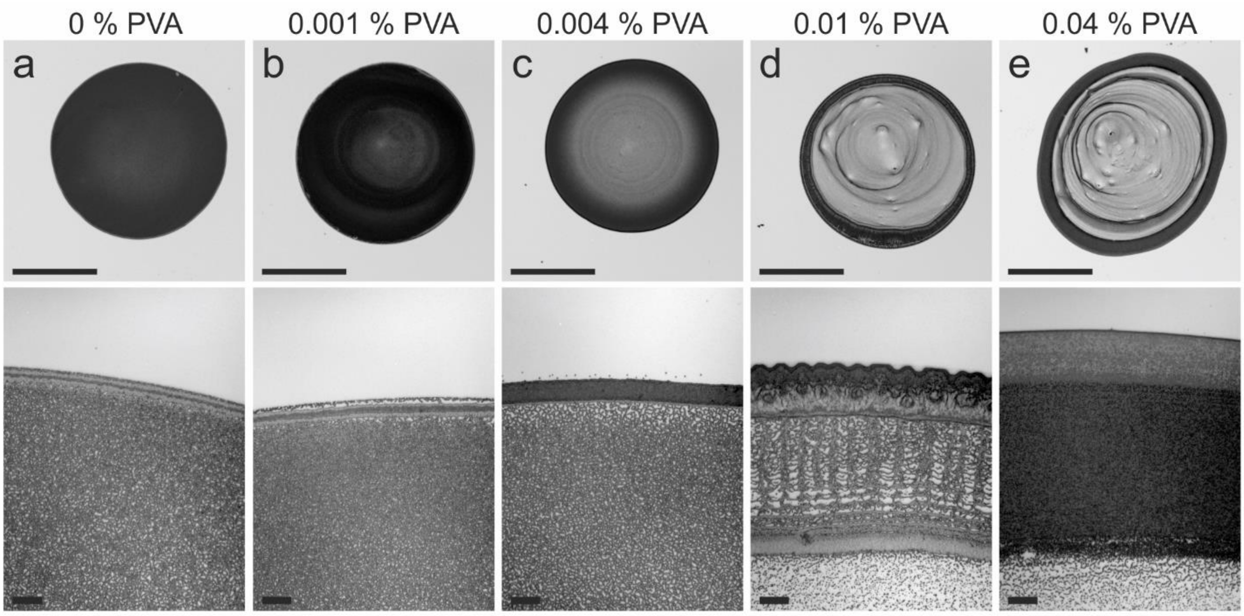

Supplementary Figure 17: Effect of adding additional “free” PVA on the drying behavior of a colloidal dispersion, coated with PVA in a previous step. The addition of free PVA induces a coffee ring. Scale bar. top: 1 mm, bottom: 20 µm.

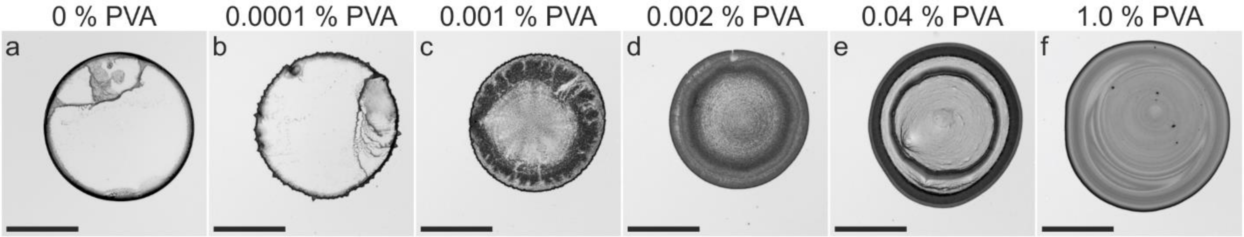

Supplementary Figure 18: Effect of adding PVA on the drying behavior of pure colloidal dispersions. Increasing the PVA content up to 0.002 wt-% leads to a more uniform drying behavior, while a further increase induces a coffee ring effect. Scale bar: 1 mm.

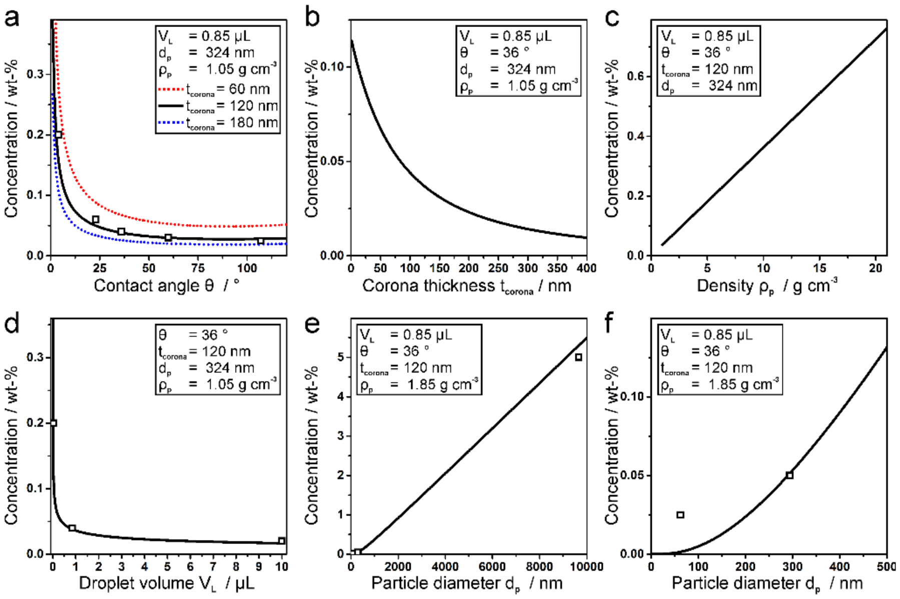

Supplementary Figure 19: Theoretical predictions of the required particle concentration to obtain a particle monolayer at the air/water interface of a drying droplet, calculated according to the model derived on the previous page. The square points correspond to the parameters used in experiments. a) Particle concentration required to form a complete monolayer as a function of contact angle for a lateral corona dimension t_{corona} of 60 nm (red dotted line), 120 nm (black line) and 180 nm (blue dotted line). A corona thickness of 120 nm best fits the experimental parameters used in Supplementary Figure 24. b) Effect of the corona thickness on the required particle concentration to obtain an interfacial monolayer for a contact angle of 36°, which approximates the experimental conditions. c) The required particle concentration scales linearly with the particle density. d) Optimal particle concentration as a function of droplet volume. The squares correspond to the experimental parameters used in Supplementary Figure 22. e,f) Optimal particle concentration as a function of particle diameter. The squares correspond to experimental parameters used in Supplementary Figure 29. The model fails for small nanoparticles as it overestimates the corona thickness and neglects the presence of bridged particles.

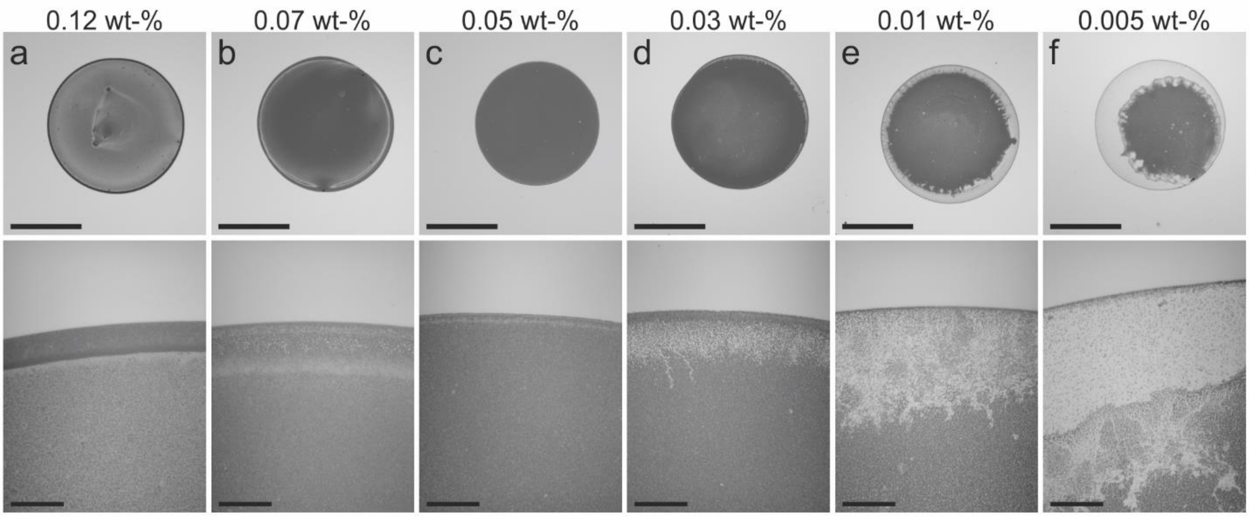

Supplementary Figure 20: Effect of particle concentration on the drying behavior of aqueous dispersions of PVA-modified PS particles. Scale bar top: 1 mm, bottom: 50 µm. Higher particle concentrations produce an accumulation of particles near the droplet edge, but also give a homogeneous interior. An optimum concentration (here: 0.05 wt-%) produces a homogeneous deposit, while smaller concentrations lead to an accumulation of particles at the droplet interior.

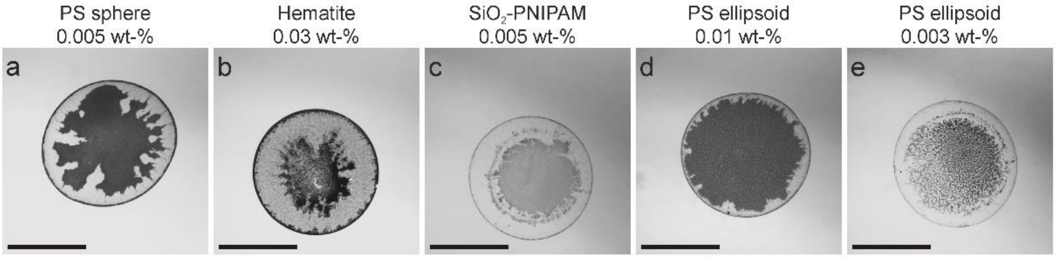

Supplementary Figure 21: Effect of a low particle concentration for different particle types on the drying behavior of polymer-modified aqueous dispersions. We observe an increase in particle concentrations towards the center of the deposit for all particle types, which we attribute to the interfacial backflow towards the apex of the droplet induce by a thermal Marangoni effect (see Supplementary Discussion 2). Scale bar: 1 mm.

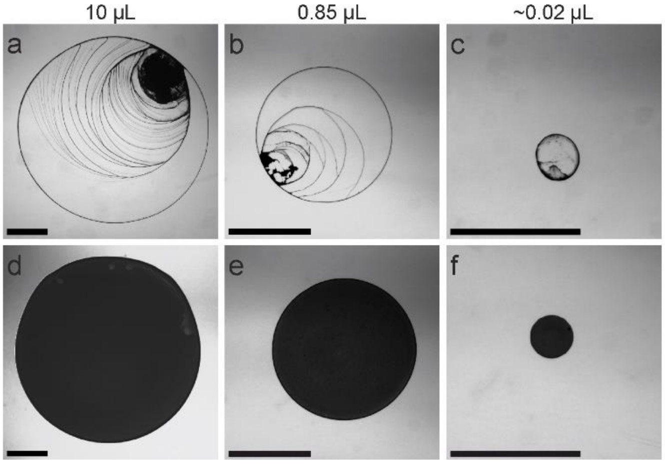

Supplementary Figure 22: Influence of the volume of the deposited PS dispersion droplets. a-c) Pure PS particle dispersion and (d-f) PVA-modified PS particle dispersion. Scale bar: 1 mm.

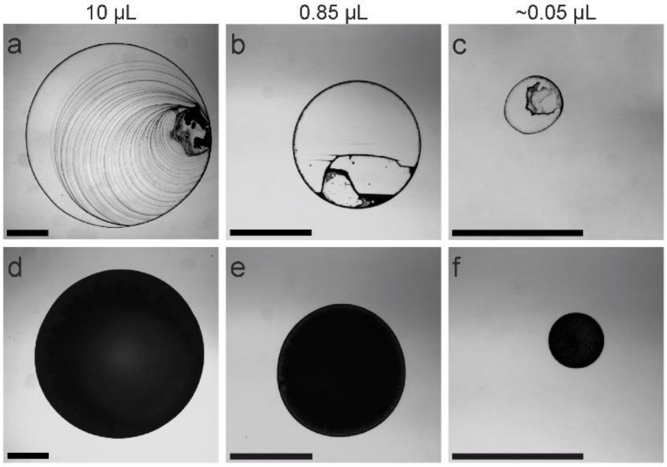

Supplementary Figure 23: Influence of the volume of deposited hematite dispersion droplets. a-c) Pure hematite particle dispersions and (d-f) PVA-modified hematite particle dispersions. Scale bar: 1 mm.

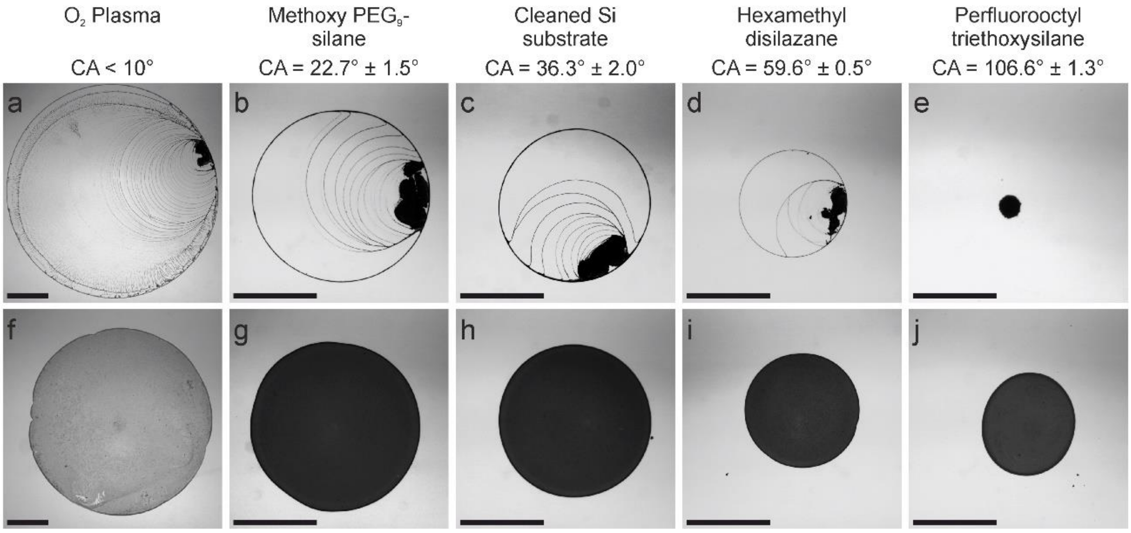

Supplementary Figure 24: Influence of the contact angle of the deposited PS dispersion droplets. a-e) Pure PS particle dispersions dried on substrates with different contact angles. f-j) PVA-modified PS particles dried on the same substrates. e) For a high contact angle of 106.6° we observe the expected dot-like deposit for pure particles due to the shrinking of the droplet during the evaporation. j) However, the PVA modified particles still dry homogeneously and the initial contact of the liquid to the substrate is preserved during the drying. Apparently, the interfacially adsorbed particle monolayer provides additional resistance against the dewetting of the droplet during the drying process. Scale bar: 1 mm.

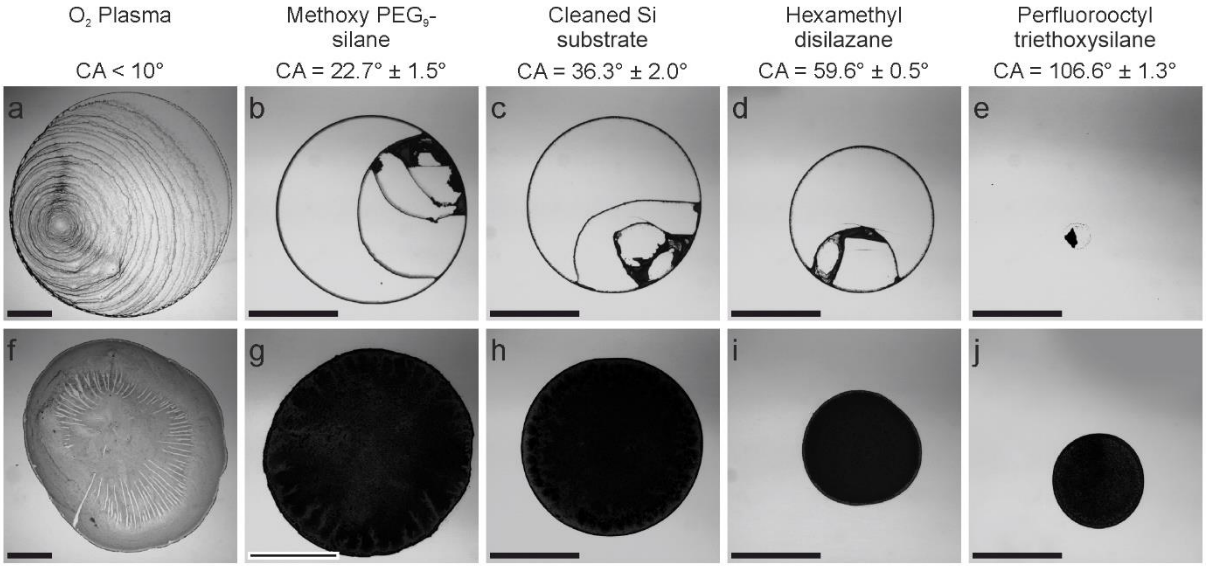

Supplementary Figure 25: Influence of the contact angle of the deposited hematite dispersion droplets. a-e) Pure hematite particle suspensions dried on substrates with different contact angles and (f-j) PVA-modified hematite particle suspensions dried on the same substrates. j) Similar to the PS particle suspension (Supplementary Figure 23), a flat drying is obtained even for high contact angles of 106.6°. Scale bar: 1 mm.

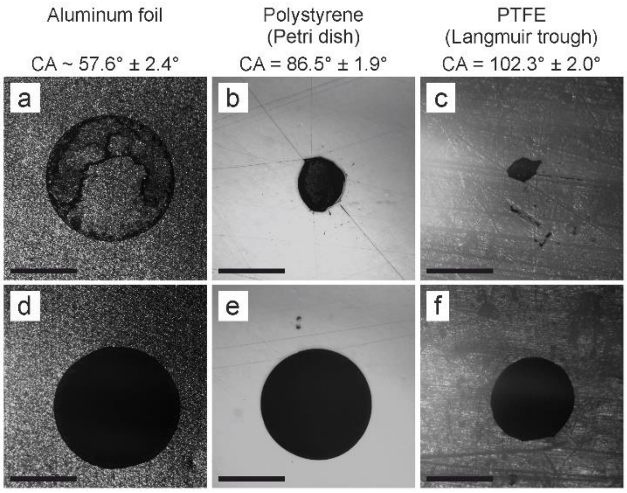

Supplementary Figure 26: Influence of the substrate material on the deposited hematite dispersion droplets. a-c) Pure hematite particle dispersions and (d-f) PVA-coated hematite particle dispersions dried on aluminum foil (a,d), polystyrene petri dish (b,e) and on a poly(tetrafluoroethylene) Langmuir trough (c,f) as substrates. While the pure particle dispersions form a coffee ring or a dot-like deposit (a-c), the PVA-coated particle dispersions form uniform deposits independent of the material type and contact angle. We note that the used substrates are not perfectly flat with roughness estimated in the µm range and they further possess scars from the machining process. The uniform deposition obtained on all substrates demonstrates the resilience of the method with regards to substrate types. Scale bar: 1 mm.

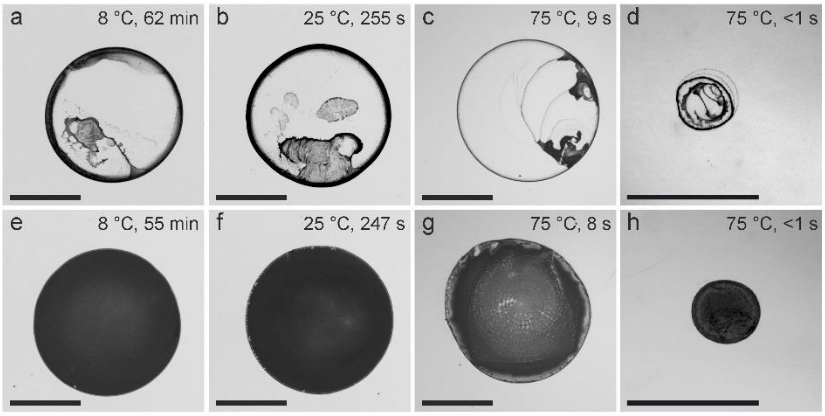

Supplementary Figure 27: Effect of drying temperature / drying rate on the drying behavior of the colloidal suspensions. a-d) Pure PS particles and (e-h) PS particles with adsorbed PVA dried at 8 °C (a,e), 25 °C (b,f) and 75 °C (c,g) as well as a small droplet volume dried at 75 °C (d,h). We observe a similar pattern after drying independent of the drying temperature / drying speed. However, the quality of the deposit decreases with increasing drying rate. Scale bar: 1 mm.

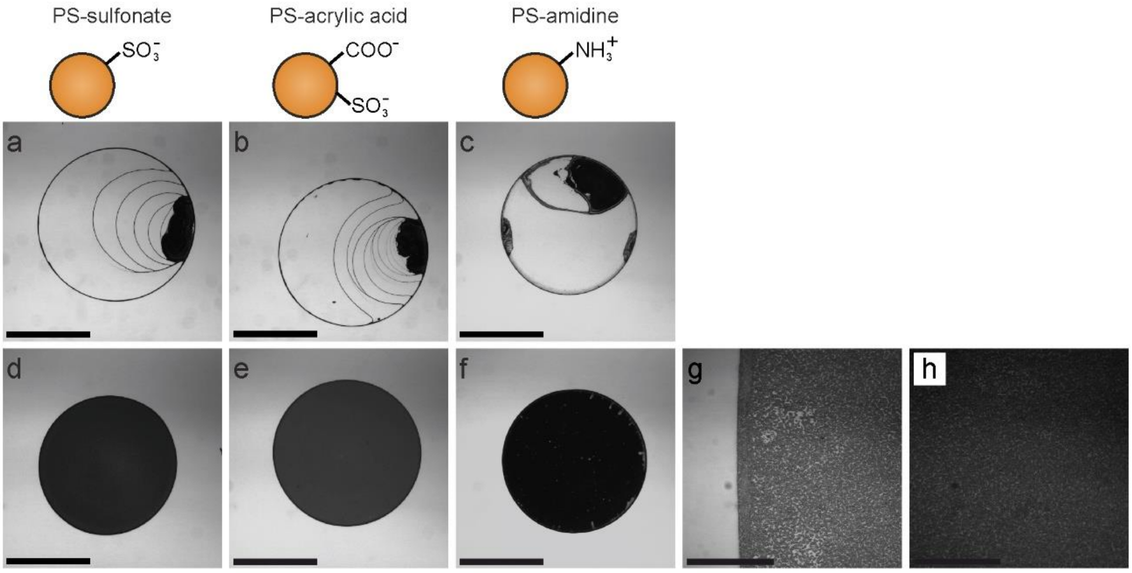

Supplementary Figure 28: Effect of particle surface chemistry and surface charge on the resulting drying behavior, examined for PS particles synthesized by surfactant-free emulsion polymerization stabilized by negatively-charged sulfonate groups from ammonium persulfate as initiator (a,d), by carboxylic acid groups introduced via acrylic acid added as comonomer (b.e) and by positively charged amidine groups from the initiator 2,2'-azobis(2-methylpropionamidine) dihydrochloride (c,f-h). The top row (a-c) shows the drying pattern of the pure particle dispersion, the bottom row (d-h) after surface modification with PVA, as in Figure 1. After PVA modification, all particle dispersions exhibited a homogenous drying pattern. a-f) Scale bar: 1 mm. Because the amidine-functionalized particles additionally contained a fluorescent dye, the dried deposit appears darker in the optical microscopy image compared to the other dispersions. Since this may hide possible multilayers, we further provide close-up optical microscopy images of (f) at the edge (g) and in the center (h) of the dried deposit, which show the presence of a particle monolayer. g,h) Scale bar: 40 µm.

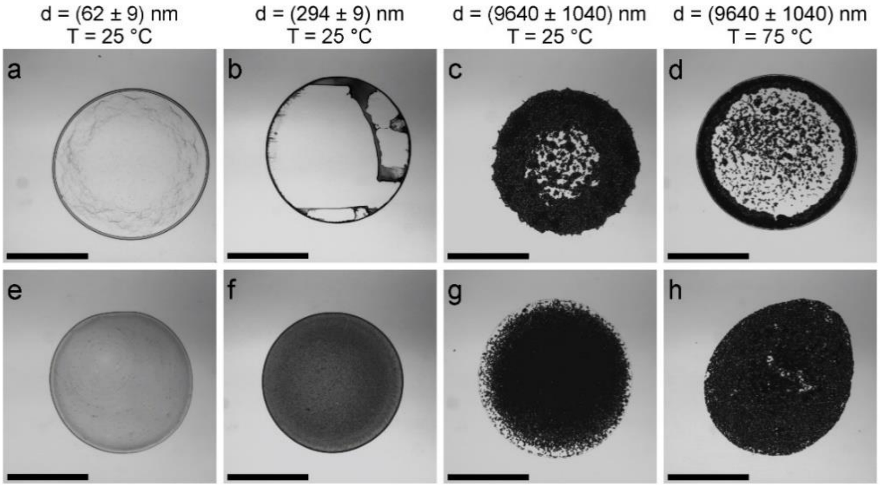

Supplementary Figure 29: Effect of silica particle size on drying behavior. a-d) Drying behavior of pure silica particles and (e-h) silica particles modified with PVA. a) Drying patterns of silica nanoparticles. Pure silica nanoparticles (d = 62 nm) undergo a coffee ring while modified particles (e) dry more homogeneous. However, in this case, as the particles are very small and their size approaches the dimensions of the polymer coils in solution, the probability of particle aggregation due to bridging increases, leading to aggregates as well as partial multilayers in the deposit. This size range of the particles therefore marks a lower boundary for a successful homogeneous drying process. Note that the color intensity is reduced due to the lower scattering contrast. c,g) Drying pattern of silica microparticles. With increasing size of the silica particles, gravity increasingly competes with the capillary flow towards the outside edge. An ill-defined drying pattern without a pronounced coffee stain results as the particle rapidly settle under the effect of gravity, even in the absence of PVA-modification. d,h) The effect of sedimentation can be circumvented by increasing the temperature to speed up the evaporation process. At 75 °C, a coffee ring is observed for the pure particles (d), while the PVA modified particles exhibit a more homogeneous drying pattern (h). Scale bar: 1 mm.

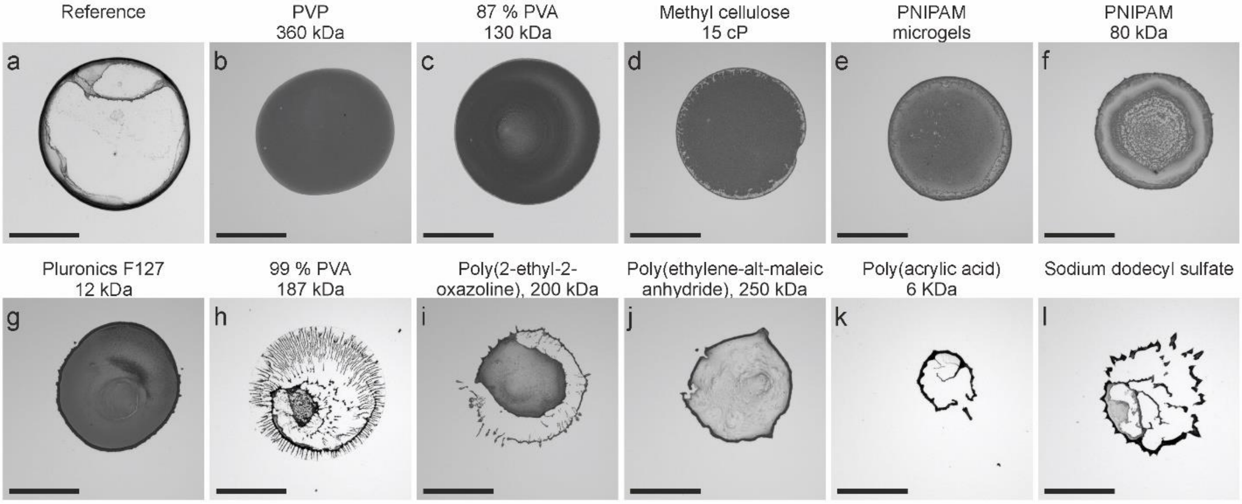

Supplementary Figure 30: Effect of commercial polymeric additives on the drying behavior of colloidal PS suspensions leading to a homogenous drying (b-f) and a coffee ring effect (h-l). Prior to the deposition, the particle/polymer suspension was cleaned 6 times by centrifugation and redispersion. a) Reference, b) PVP, c) 87 % hydrolyzed PVA, d) methyl cellulose, e) PNIPAM microgels, f) PNIPAM, g) Pluronics, h) 99 % hydrolyzed PVA, i) poly(2-ethyl-2-oxazoline), j) poly(ethylene-alt-maleic anhydride), k) poly(acrylic acid) and l) sodium dodecyl sulfate. Scale bar: 1 mm.

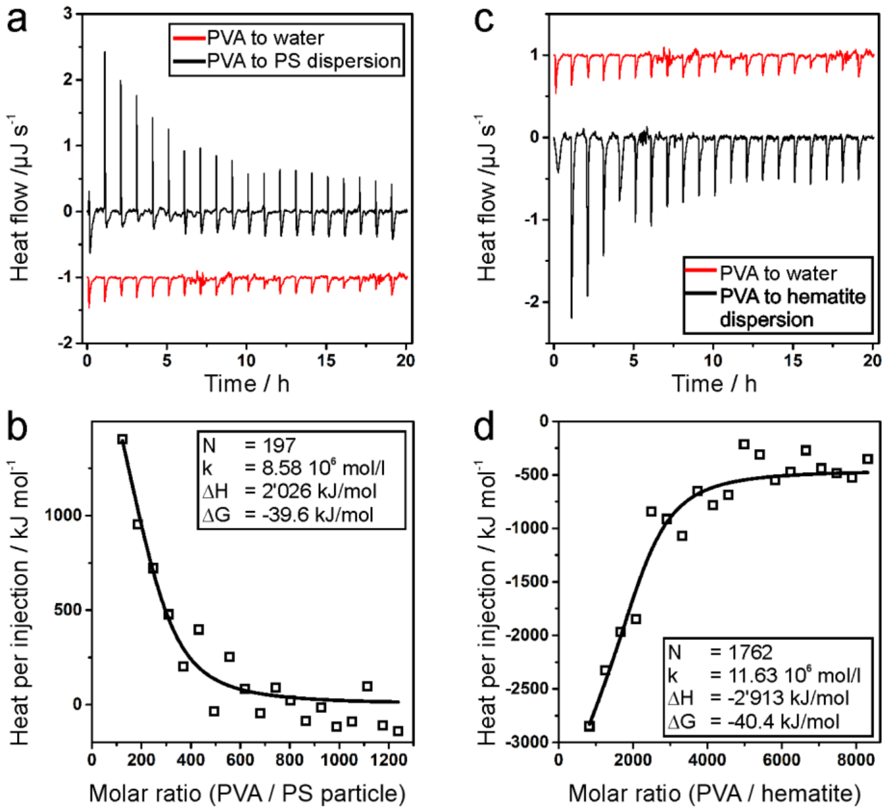

Supplementary Figure 31: Isothermal titration calorimetry of PVA added to a PS particle dispersion (a,b) and to a hematite particle suspension (c,d). a,c) Baseline-corrected heat rate of the titration for PVA added to water (red lines) and PVA added to the particle dispersions (black lines). The red line has been shifted by 1 µJ/s for visualization purposes. b,d) Integrated normalized heats from each titration step together with a fit corresponding to an independent binding model.[1–3]

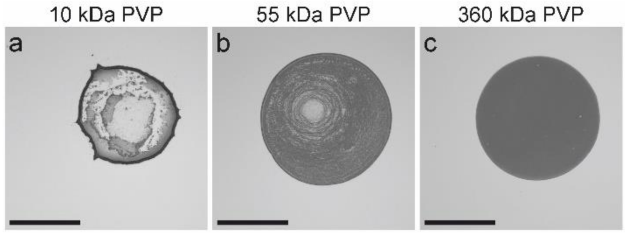

Supplementary Figure 32: Effect of polymer additive chain length. With increasing chain length and thus increasing hydrodynamic diameter D_H (Supplementary Figure 4), the drying behavior of the suspended particles is more homogenous. Scale bar: 1 mm.

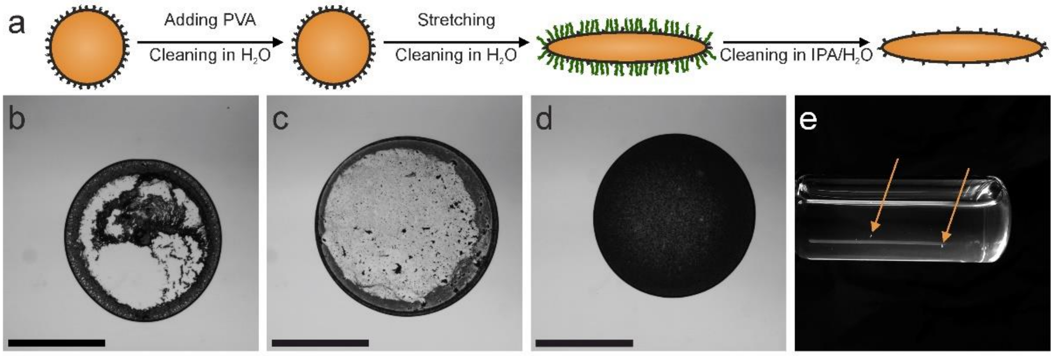

Supplementary Figure 33: Drying behavior of sterically stabilized PS particles prepared by dispersion polymerization with mono vinyl-terminated poly(ethylene glycol) (PEG, 2 kDa) as comonomer. The particles were stretched into elliptical shapes similar to the charge-stabilized particles in Figure 2. a) Schematic illustration of the particles, their shape and their hypothesized surface functionality. b) Sterically stabilized PS particles form a coffee ring, although less defined compared to the charge stabilized PS particles. c) We added PVA to the particle dispersion to induce physisorption, similar as in Figure 1. After removing non-adsorbed PVA by centrifugation and redispersion in water, the dispersions exhibited a coffee ring upon drying, in contrast to the case of Figure 1. We hypothesize that these PEG chains hinder the adsorption of the PVA chains to the particle surface. The system therefore acts similar to the unmodified case. d) We subsequently thermo-mechanically stretched the sterically stabilized PS particles into an elliptical shape by embedding in a PVA foil. After removing the PVA by centrifugation and redispersion in water, a homogeneous drying was observed. We hypothesize that the increase in surface area of the particles upon stretching creates unprotected areas onto which the longer PVA chains can adsorb. These adsorbed PVA chains then induce the homogeneous drying behavior. e) Upon removal of the adsorbed PVA chains by centrifugation and redispersion in an IPA/H_2O mixture, the ellipsoidal particles aggregated due to the insufficient steric stabilization, corroborating with the hypothesis that free surface is formed upon stretching. The aggregates are indicated by the orange arrows. Scale bar: 1 mm.

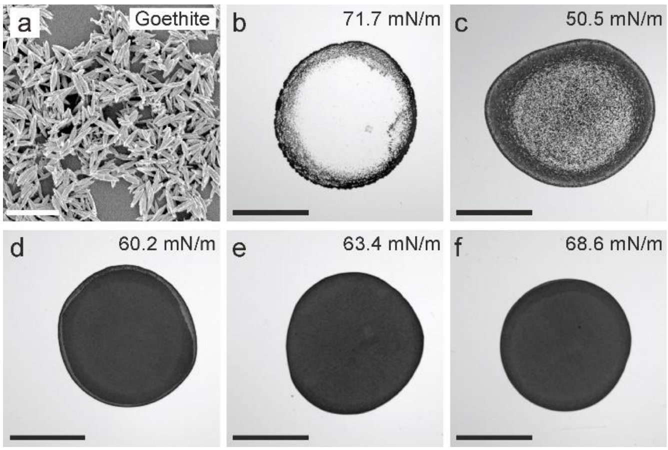

Supplementary Figure 34: Uniform drying patterns of goethite pigment particle dispersions. a) SEM images of the respective pure particles. Scale bar: 1 µm. b-f) Optical microscopy images of the corresponding dried dispersion droplets of pure particles (b) and particles with PVA coating (c-f) without removal of excess PVA (c) and after removing excess PVA by one (d), two (e) and three (f) centrifugation and redispersion cycles. Scale bar: 1 mm.

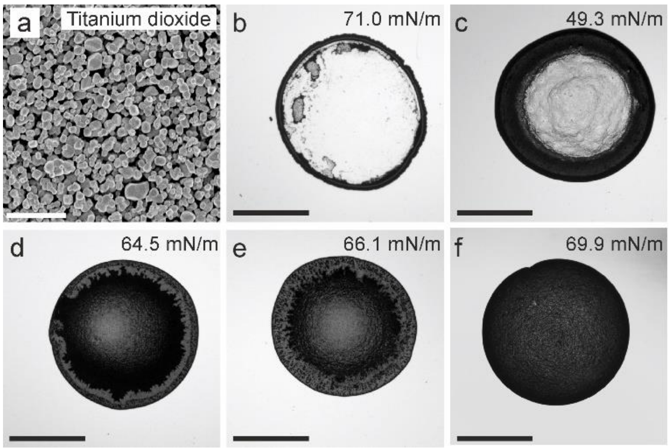

Supplementary Figure 35: Uniform drying patterns of titanium oxide pigment particle dispersions. a) SEM images of the respective pure particles. Scale bar: 1 µm. b-f) Optical microscopy images of the corresponding dried dispersion droplets of pure particles (b) and particles with PVA coating (c-f) without removal of excess PVA (c) and after removing excess PVA by one (d), two (e) and three (f) centrifugation and redispersion cycles. Scale bar: 1 mm.

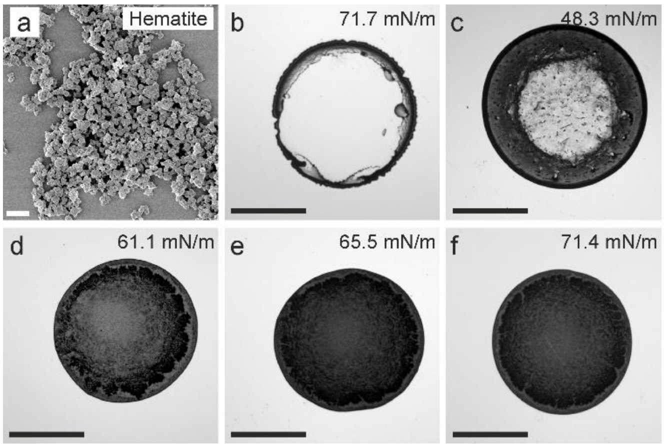

Supplementary Figure 36: Uniform drying patterns of hematite pigment particle dispersions. a) SEM images of the respective pure particles. Scale bar: 1 µm. b-f) Optical microscopy images of the corresponding dried dispersion droplets of pure particles (b) and particles with PVA coating (c-f) without removal of excess PVA (c) and after removing excess PVA by one (d), two (e) and three (f) centrifugation and redispersion cycles. Scale bar: 1 mm.

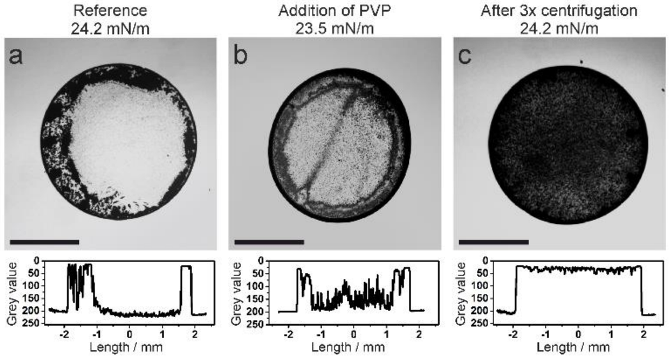

Supplementary Figure 37: Drying behavior of hematite particles dispersed in butanol. Drying behavior of (a) a reference colloidal dispersion, (b) the same dispersion with addition of PVP (weight ratio 1:1) and (c) after removal of non-adsorbed PVP, in which case a homogeneous drying behavior was observed. The surface-modification of the particles has been carried out in butanol, similar to aqueous modification shown in Figure 1. Hematite particles were chosen over PS particles, as PS particles would dissolve in many polar solvents. Prior to the drying experiment, the silica substrate was rendered omniphobic by surface treatment with fluorosilanes to ensure that a well-defined sessile droplet formed for all solvents (the contact angle on unfunctionalized substrate was < 10° for butanol, leading to spreading of the dispersion). In (a-c) top image: Optical micrograph of the dried dispersions. Scale bar: 1 mm. Bottom: Cross-sectional grey value distribution after drying. The surface tension of the particle dispersions has been measured using the Wilhelmy method.

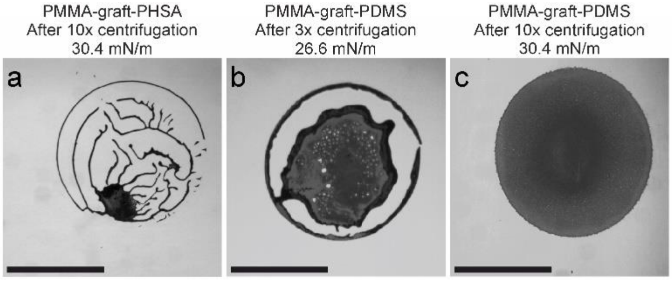

Supplementary Figure 38: Drying behavior of hydrophobic poly(methyl methacrylate) (PMMA) particles dispersed in decalin. The substrate was rendered omniphobic with fluorosilanes to ensure the formation of a sessile droplet. a) As reference particle system we chose PMMA particles stabilized with poly(12-hydroxy steric acid) (PHSA), which form multiple coffee rings in agreement with previous work.[23–25] Note, however, that some studies using larger particles report uniform drying for this system.[15] b,c) PMMA particles stabilized by the copolymerization of surface-active, monovinyl-terminated poly(dimethyl siloxane) (PDMS) macromonomers. b) Insufficient cleaning of the particles combined with a lowered surface tension still leads to a coffee ring. c) After removal of “free” PDMS and potentially other oligomeric impurities, a homogeneous drying pattern is observed. This behavior indicates that the low surface energy of the grafted PDMS chains at the particle surface causes interfacial adsorption of the PMMA particles. Scale bar: 1 mm.

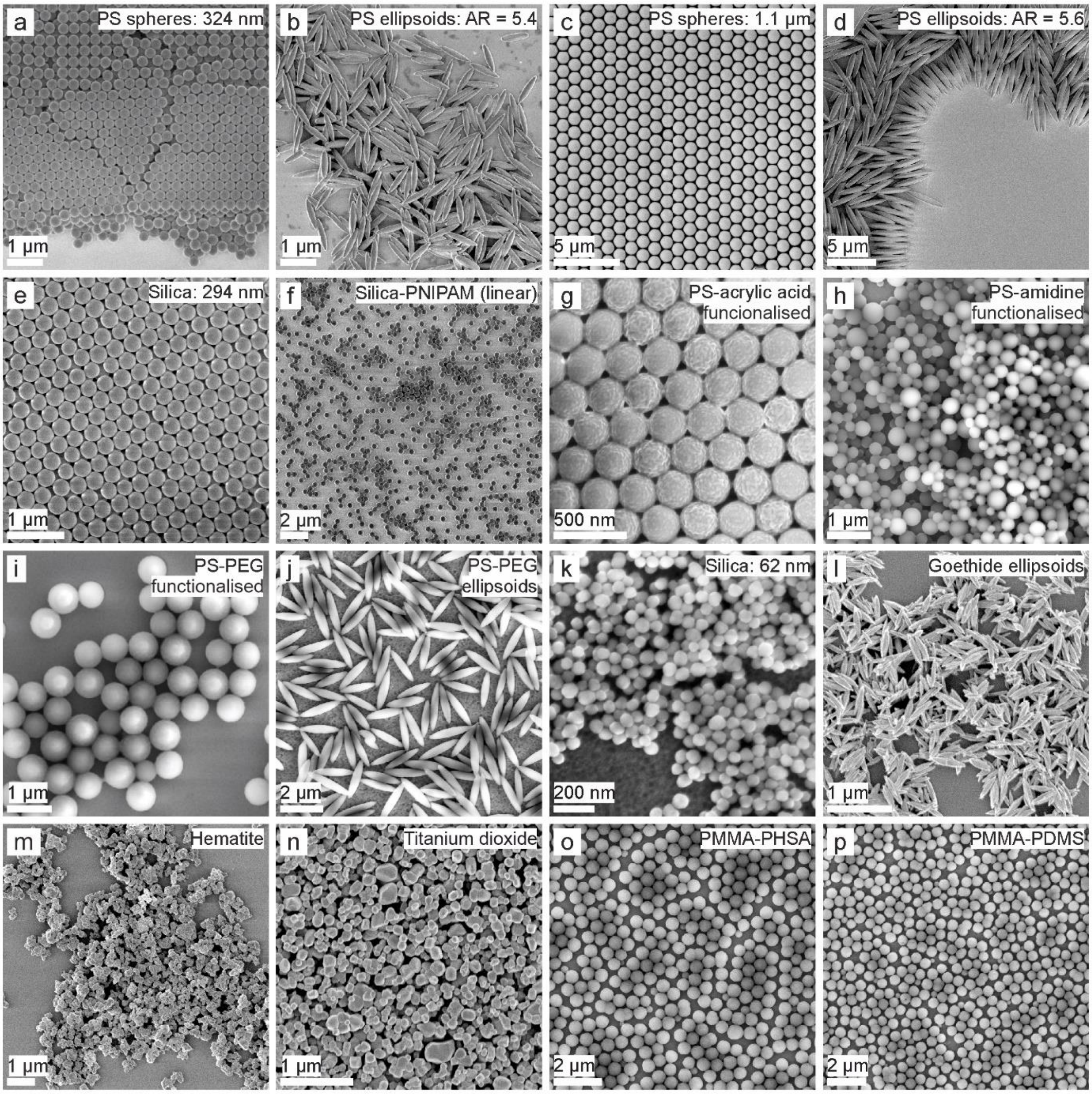

Supplementary Figure 39: SEM images of the different particle systems used in this study.

Supplementary Table: Experimental details

Supplementary Table 1: Experimental details for each of the presented dried droplets.

		Particle type	Particle diameter [nm]	Particle concen-tration [wt-%]	Polymer coating	Droplet volume [µL]]	Comments
Figure 1	c,d) left	PS	324	0.04	-	0.85	
	c,d) middle	PS	324	0.04	PVA 130 kDa	0.85	Contains non-adsorbed PVA (particle /PVA weight ratio 1:1)
	c,d) right	PS	324	0.04	PVA 130 kDa	0.85	
	e) left	PS	500	0.04	-	0.85	Fluorescent
	e) middle	PS	500	0.04	PVA 130 kDa	0.85	Fluorescent, contains non-adsorbed PVA
	e) right	PS	500	0.04	PVA 130 kDa	0.85	Fluorescent
Figure 2	a)	PS	1050 / 194	0.04	PVA 130 kDa	1.5	Ellipsoids
	d)	PS	1050 / 194	0.04	-	1.5	Ellipsoids
	b)	PS	324	0.04	PVA 130 kDa	1.5	
	e)	PS	324	0.04	-	1.5	
	c)	Silica	294	0.03	grafted PNIPAM	0.85	
	f)	Silica	294	0.04	-	0.85	
Figure 3	top	PS	1100	0.1	-	1.5	
	middle	PS	1100	0.1	PVA 130 kDa	1.5	
	bottom	PS	3700 / 720	0.1	PVA 130 kDa	1.5	Ellipsoids
	purple	PS	1100	0.1	PVA 60 kDa	1.5	
	green	PS	1100	0.1	PVA 130 kDa	1.5	Contains non-adsorbed PVA (particle /PVA weight ratio 1:1)
Figure 4	a-d)	PS	2000 / 324	0.05	-	1.0	0.045 wt% PS particles (d = 324 nm) and 0.005 wt-% fluorescent tracer particles (d = 2 µm)
	e-h)	PS	2000 / 324	0.05	PVA 130 kDa	1.0	0.045 wt% PS particles (d = 324 nm) and 0.005 wt-% fluorescent tracer particles (d = 2 µm). Both particle species were modified with PVA.
Figure 5	b)	Goethite		0.14	-	0.85	
	c)	Titanium Oxide		0.14	-	0.85	
	d)	Hematite		0.14	-	0.85	
	e)	Goethite		0.14	PVA 130 kDa	0.85	
	f)	Titanium Oxide		0.16	PVA 130 kDa	0.85	
	g)	Hematite		0.14	PVA 130 kDa	0.85	
	h)	Hematite		0.1		1.5	Substrate: perfluorooctyl silane, Particles dispersed in DMF, CA = 73°
	i)	Hematite		0.11		1.5	Substrate: perfluorooctyl silane, Particles dispersed in heptanol, CA = 66°, dried at 75 °C
	j)	Hematite		0.11		1.5	Substrate: perfluorooctyl silane, Particles dispersed in butanol, CA = 59°
	k)	Hematite		0.13		1.5	Substrate: perfluorooctyl silane, Particles dispersed in ethanol, CA = 41°
	l)	Hematite		0.1	PVA 130 kDa	1.5	Substrate: perfluorooctyl silane, Particles dispersed in DMF, CA = 73°
	m)	Hematite		0.11	PVA 130 kDa	1.5	Substrate: perfluorooctyl silane, Particles dispersed in heptanol, CA = 66°, dried at 75 °C
	n)	Hematite		0.11	PVA 130 kDa	1.5	Substrate: perfluorooctyl silane, Particles dispersed in butanol, CA = 59°
	o)	Hematite		0.13	PVA 130 kDa	1.5	Substrate: perfluorooctyl silane, Particles dispersed in ethanol, CA = 41°
Supporting Figures							
Supplementary Figure 1	a)	PS	324	0.04	-	0.85	
	b)	PS	324	0.04	PVA 130 kDa	0.85	PVA ratio 1:1 (particle /PVA weight ratio 1:1)
	c)	PS	324	0.04	PVA 130 kDa	0.85	1x cent.
	d)	PS	324	0.04	PVA 130 kDa	0.85	2x cent.

	e)	PS	324	0.04	PVA 130 kDa	0.85	3x cent.
Supplementary Figure 3	b-e)	PS	324	0.04	-	1.5	
	f-i)	PS	324	0.04	PVA 130 kDa	1.5	
Supplementary Figure 8		PS	3700 / 720	0.1	PVA 130 kDa	1.5	Ellipsoids
Supplementary Figure 9	a-c)	PS	3700 / 720	0.05	PVA 130 kDa	0.85	Ellipsoids
Supplementary Figure 10		PS	1100	0.1	PVA 130 kDa	1.5	
Supplementary Figure 11	a-e)	PS	1100	0.1	-	0.85	
	f-j)	PS	1100	0.1	PVA 130 kDa	0.85	
Supplementary Figure 12		PS	2000 / 324	0.05	-	1.0	0.045 wt% PS particles (d = 324 nm) and 0.005 wt-% fluorescent tracer particles (d = 2 µm)<br>CA = 39 °
Supplementary Figure 13		PS	2000 / 324	0.05	-	1.0	0.045 wt% PS particles (d = 324 nm) and 0.005 wt-% fluorescent tracer particles (d = 2 µm)<br>CA = 39 °
Supplementary Figure 14		PS	2000 / 324	0.05	PVA 130 kDa	1.0	0.045 wt% PS particles (d = 324 nm) and 0.005 wt-% fluorescent tracer particles (d = 2 µm). Both particle species were modified with PVA.<br>CA = 39 °
Supplementary Figure 15		PS	2000 / 324	0.05	PVA 130 kDa	1.0	0.045 wt% PS particles (d = 324 nm) and 0.005 wt-% fluorescent tracer particles (d = 2 µm). Both particle species were modified with PVA.<br>CA = 39 °
Supplementary Figure 16	a-e	PS	324	0.04	PVA 130 kDa	1.5	
	f-j	PS	1050 / 194	0.04	PVA 130 kDa	1.5	Ellipsoids
	k-o	Silica	294	0.03	grafted PNIPAM	0.85	
	p-s	PS	324	0.04	-	1.5	
Supplementary Figure 17	a-e)	PS	324	0.04	PVA 130 kDa	0.85	addition of "free" PVA
Supplementary Figure 18	a)	PS	324	0.04	-	0.85	
	b-f)	PS	324	0.04	PVA 130 kDa	0.85	Contains non-adsorbed PVA
Supplementary Figure 20	a-f)	PS	324	-	PVA 130 kDa	0.85	Change in particle concentration
Supplementary Figure 21	a)	PS	324	0.005	PVA 130 kDa	0.85	
	b)	Hematite		0.03	PVA 130 kDa	0.85	
	c)	Silica	294	0.005	grafted PNIPAM	0.85	
	d)	PS	1050 / 194	0.01	PVA 130 kDa	0.85	Ellipsoids
	e)	PS	1050 / 194	0.003	PVA 130 kDa	0.85	Ellipsoids
Supplementary Figure 22	a)	PS	324	0.02	-	10	
	b)	PS	324	0.04	-	0.85	
	c)	PS	324	0.2	-	~0.02	Drop volume estimated
	d)	PS	324	0.02	PVA 130 kDa	10	
	e)	PS	324	0.04	PVA 130 kDa	0.85	
	f)	PS	324	0.2	PVA 130 kDa	~0.02	Drop volume estimated
Supplementary Figure 23	a)	Hematite		0.07	-	10	
	b)	Hematite		0.14	-	0.85	
	c)	Hematite		0.6	-	~0.05	Drop volume estimated
	d)	Hematite		0.07	PVA 130 kDa	10	
	e)	Hematite		0.14	PVA 130 kDa	0.85	
	f)	Hematite		0.6	PVA 130 kDa	~0.05	Drop volume estimated
Supplementary Figure 24	a)	PS	324	0.2	-	0.85	Substrate plasma treated, CA < 10°
	b)	PS	324	0.06	-	0.85	Substrate: mPEG9-silane, CA = 23°
	c)	PS	324	0.04	-	0.85	Substrate cleaned as usual, CA = 36°
	d)	PS	324	0.03	-	0.85	Substrate: HDMS, CA = 60°

	e)	PS	324	0.025	-	0.85	Substrate: perfluorooctyl silane, CA = 107°
	f)	PS	324	0.2	PVA 130 kDa	0.85	Substrate plasma treated, CA < 10°
	g)	PS	324	0.06	PVA 130 kDa	0.85	Substrate: mPEG9-silane, CA = 23°
	h)	PS	324	0.04	PVA 130 kDa	0.85	Substrate cleaned as usual, CA = 36°
	i)	PS	324	0.03	PVA 130 kDa	0.85	Substrate: HDMS, CA = 60°
	j)	PS	324	0.025	PVA 130 kDa	0.85	Substrate: perfluorooctyl silane, CA = 107°
Supplementary Figure 25	a)	Hematite		0.65	-	0.85	Substrate plasma treated, CA < 10°
	b)	Hematite		0.2	-	0.85	Substrate: mPEG9-silane, CA = 23°
	c)	Hematite		0.14	-	0.85	Substrate cleaned as usual, CA = 36°
	d)	Hematite		0.1	-	0.85	Substrate: HDMS, CA = 60°
	e)	Hematite		0.09	-	0.85	Substrate: perfluorooctyl silane, CA = 107°
	f)	Hematite		0.65	PVA 130 kDa	0.85	Substrate plasma treated, CA < 10°
	g)	Hematite		0.2	PVA 130 kDa	0.85	Substrate: mPEG9-silane, CA = 23°
	h)	Hematite		0.14	PVA 130 kDa	0.85	Substrate cleaned as usual, CA = 36°
	i)	Hematite		0.1	PVA 130 kDa	0.85	Substrate: HDMS, CA = 60°
	j)	Hematite		0.09	PVA 130 kDa	0.85	Substrate: perfluorooctyl silane, CA = 107°
Supplementary Figure 26	a)	Hematite		0.1	-	0.85	Substrate: aluminum foil, CA = 58 °
	b)	Hematite		0.095	-	0.85	Substrate: PS petri dish, CA = 87 °
	c)	Hematite		0.09	-	0.85	Substrate: PTFE Langmuir trough, CA = 102 °
	d)	Hematite		0.1	PVA 130 kDa	0.85	Substrate: aluminum foil, CA = 58 °
	e)	Hematite		0.095	PVA 130 kDa	0.85	Substrate: PS petri dish, CA = 87 °
	f)	Hematite		0.09	PVA 130 kDa	0.85	Substrate: PTFE Langmuir trough, CA = 102 °
Supplementary Figure 27	a)	PS	324	0.04	-	0.85	Evaporated at 8 °C
	b)	PS	324	0.04	-	0.85	Evaporated at 25 °C
	c)	PS	324	0.04	-	0.85	Evaporated at 75 °C
	d)	PS	324	0.15	-	~0.05	Evaporated at 75 °C
	e)	PS	324	0.04	PVA 130 kDa	0.85	Evaporated at 8 °C
	f)	PS	324	0.04	PVA 130 kDa	0.85	Evaporated at 25 °C
	g)	PS	324	0.04	PVA 130 kDa	0.85	Evaporated at 75 °C
	fh	PS	324	0.15	PVA 130 kDa	~0.05	Evaporated at 75 °C
Supplementary Figure 28	a)	PS	324	0.04	-	0.85	Sulfonate stabilized PS
	b)	PS	270	0.03	-	0.85	Acrylic acid stabilized PS
	c)	PS	335	0.04	-	0.85	Amidine stabilized PS
	d)	PS	324	0.04	PVA 130 kDa	0.85	Sulfonate stabilized PS
	e)	PS	270	0.03	PVA 130 kDa	0.85	Acrylic acid stabilized PS
	f)	PS	335	0.04	PVA 130 kDa	0.85	Amidine stabilized PS
	g)	PS	335	0.04	PVA 130 kDa	0.85	Amidine stabilized PS – 50x objective
	h)	PS	335	0.04	PVA 130 kDa	0.85	Amidine stabilized PS – 50x objective
Supplementary Figure 29	a)	Silica	62	0.025		0.85	
	b)	Silica	294	0.05		0.85	
	c)	Silica	9640	5.0		0.85	
	d)	Silica	9640	5.0		0.85	Dried at 75 °C
	e)	Silica	62	0.025	PVA 130 kDa	0.85	
	f)	Silica	294	0.05	PVA 130 kDa	0.85	
	g)	Silica	9640	5.0	PVA 130 kDa	0.85	
	h)	Silica	9640	5.0	PVA 130 kDa	0.85	Dried at 75 °C
Supplementary Figure 30	a)	PS	324	0.04	-	0.85	
	b-l)	PS	324	0.04	various polymers	0.85	
Supplementary Figure 32	a)	PS	324	0.04	PVP 360 kDa	0.85	
	b)	PS	324	0.04	PVP 55 kDa	0.85	
	c)	PS	324	0.04	PVP 10 kDa	0.85	

Supplementary Figure 33	b)	PS-PEG	647	0.11		0.85	
	c)	PS-PEG	647	0.11	PVA 130 kDa	0.85	
	d)	PS-PEG	1865/371	0.11	PVA 130 kDa	0.85	Ellipsoids
	e)	PS-PEG	1865/371	-			Ellipsoids, IPA/H_2O cleaned, aggregated
Supplementary Figure 34	b)	Goethite		0.14	-	0.85	
	c)	Goethite		0.14	PVA 130 kDa	0.85	PVA ratio 1:1 (particle /PVA weight ratio 1:1)
	d)	Goethite		0.14	PVA 130 kDa	0.85	1x cent.
	e)	Goethite		0.14	PVA 130 kDa	0.85	2x cent.
	f)	Goethite		0.14	PVA 130 kDa	0.85	3x cent.
Supplementary Figure 35	b)	Titanium Oxide		0.14	-	0.85	
	c)	Titanium Oxide		0.14	PVA 130 kDa	0.85	PVA ratio 1:1 (particle /PVA weight ratio 1:1)
	d)	Titanium Oxide		0.14	PVA 130 kDa	0.85	1x cent.
	e)	Titanium Oxide		0.14	PVA 130 kDa	0.85	2x cent.
	f)	Titanium Oxide		0.16	PVA 130 kDa	0.85	3x cent.
Supplementary Figure 36	b)	Hematite		0.14	-	0.85	
	c)	Hematite		0.14	PVA 130 kDa	0.85	PVA ratio 1:1 (particle /PVA weight ratio 1:1)
	d)	Hematite		0.14	PVA 130 kDa	0.85	1x cent.
	e)	Hematite		0.14	PVA 130 kDa	0.85	2x cent.
	f)	Hematite		0.14	PVA 130 kDa	0.85	3x cent.
Supplementary Figure 37	a)	Hematite		0.11		1.5	Substrate: perfluorooctyl silane,<br>Particles dispersed in butanol, CA = 59°
	b)	Hematite		0.11	PVP 360 kDa	1.5	Substrate: perfluorooctyl silane,<br>Particles dispersed in butanol, CA = 59°<br>Contains non-adsorbed PVP. Particle / PVP ratio 1:1
	c)	Hematite		0.11	PVP 360 kDa	1.5	Substrate: perfluorooctyl silane,<br>Particles dispersed in butanol, CA = 59°<br>3x cent.
Supplementary Figure 38	a)	PMMA	531	0.08	Grafted PHSA	~1	Substrate: perfluorooctyl silane,<br>Particles dispersed in decalin, CA = 66°, dried at 75 °C<br>10x cent.
	b)	PMMA	534	0.08	Grafted PDMS	~1	Substrate: perfluorooctyl silane,<br>Particles dispersed in decalin, CA = 66°, dried at 75 °C.<br>3x cent.<br>Contains non-adsorbed PDMS
	c)	PMMA	534	0.08	Grafted PDMS	~1	Substrate: perfluorooctyl silane,<br>Particles dispersed in decalin, CA = 66°, dried at 75 °C<br>10x cent.

Supplementary Discussion 1: Droplet drying behavior

In addition to the change in drying behavior, we observe a change in the pinning/depinning behavior and the contact angle hysteresis for PVA-modified particle dispersions compared to the pure reference dispersion (Supplementary Figure 3). A drying droplet of both dispersions initially pins at its maximum extension due to the substrate roughness, chemical inhomogeneities and via trapping of the colloidal particles by immersion capillary forces at the meniscus. For the unmodified reference sample, the initial pinning is rapidly enforced by the accumulation of particles at the drying edge, which produces a pronounced coffee stain (Supplementary Figure 3b). After sufficient water evaporation, the droplet depins and rapidly moves inwards until repinning occurs, as shown in Supplementary Figure 3c-e and Supplementary Movie 1,4,5. The repinning event produces a new drying ring within the initial droplets perimeter (Supplementary Figure 3d,e). The rapid pinning/depinning events are also evident in the area of the droplet over time, which shows sudden drops and a step-wise decrease (Supplementary Figure 3a).

In the case of PVA-modified particle dispersions, the initial pinning event is only followed by the formation of a monolayer of non-close packed particles at the drop edge (see also Figure 1 and Figure 3), instead of the pronounced, close packed and multilayered particle agglomerates seen for the pure particles. Thus, less energy is required for de-pinning, which therefore occurs continuously. Upon depining, the interfacial particle monolayer formed at the air/water interface is deposited to the substrate (Supplementary Figure 3,f-i). As a result, the droplet diameter decreases homogeneously over time (Supplementary Figure 3a and Supplementary Movie 2,3).

Supplementary Discussion 2: Flow analysis within drying droplets:

We characterize the flow within the drying dispersion droplets by tracking the movement of fluorescent tracer particles added to the dispersions. The experimental set-up is adapted from Marín *et al.*,[9] whereby fluorescent microscopy images are taken every 0.5 s near the substrate and 30 µm above it using a 10× / NA = 0.3 objective. The pure dispersion consists of a mixture of 0.045 wt% non-fluorescent PS particles (d = 324 nm) and 0.005 wt-% green fluorescent PS tracer particles (d = 2 µm). The PVA-modified dispersion consists of the same ratio between non-fluorescent and tracer particles. To ensure that all particles in the dispersion behaved similarly, the tracer particles are also modified with PVA, similar to the non-fluorescent species.

The particle movement within the focal plane (depth of field ≈ 8.5 µm) is then analyzed by finding the position of each particle using ImageJ software employing the thresholding method combined with a custom-written particle tracking and analysis Matlab software (Supplementary Movie 12-15, Supplementary Figure 12-15). The position of the particles in the current frame is indicated by a cyan circle overlayed onto the original image. In addition, the particle positions of the previous 50 frames (previous 25 s) are plotted in a color gradient from bright to dark blue to visualize the trajectory of the tracer particles (Supplementary Movie 12-15, Supplementary Figure 12-15 (c-e, middle)). We note that with the chosen settings of the threshold method we are able to reliably track the majority of the particles within the depth of field. However, with the chosen threshold parameter settings, the method fails and no particles are detected if the particles are in too close contact, which for example occurs when either two particles cross path, when particles accumulate within the coffee ring or after capillary forces induce aggregation after drying.

The fluorescence images of the deposit after drying (Supplementary Figure 12-15 (a)) reveal a clear coffee ring for the pure particles and a flat, homogeneous drying for the PVA-modified particles. Therefore, we conclude that the addition of larger tracer particles does not affect the drying behavior. For both modified and pure particle dispersions, we extract the average in-plane particle velocity in the course of the drying process from the particle trajectories shown in Supplementary Movie 12-15, which is plotted in Supplementary Figure 12-15 (b). Note that this average particle velocity does not distinguish the direction in which the individual particles travel. This analysis reveals that for both types of dispersions, the average particle velocity close to the substrate (focal plane 4 µm above the substrate) increases in the course of the drying process, in agreement with previous publications.[9,10] With increasing distance to the substrate (focal plane in 30µm distance), the average in-plane particle velocity measured during the first half of the drying is larger, in agreement with previous work,[11] with a similar increase over time during the first two thirds of the evaporation. Notably, the particle velocity is higher for pure particles compared to PVA-coated particles.

Next, we investigate the direction of particle movement by statistically analyzing the movement of all particles at three different times: at the beginning, in the middle, and towards the end of the drying process (Supplementary Figure 12-15 (c-e)). We convert the particle position into polar coordinates, whereby the center of the drying droplet is estimated by fitting a circle to the contact line of the droplet segment and analyze the movement in a histogram that shows the change in radial component over 5 s for all detected particles. Positive values refer to an outward flow and negative values to an inward flow (Supplementary Figure 12-15 (c-e, bottom)). Combined with the snapshots of the particle trajectories (Supplementary Figure 12-15 (c-e, middle)), we conclude that for all samples, the flow is predominantly radial, either outwards or inwards. In addition, the trajectory snapshots reveal that particles close to the droplet edge move substantially faster compared to particles near the center of the droplet, which explains the broad distribution in the histogram (Supplementary Figure 12-15 (c-e, bottom)) and the large standard deviation in Supplementary Figure 12-15 (b), which averages over all movements.

The relative position of the focal plane compared to the height of the drying droplet changes during evaporation, which is schematically illustrated in Supplementary Figure 12-15 (c-e, top). At the beginning of the measurement, the focal plane mainly captures the particle behavior in the bulk of the droplet, whereas at later stages, due to water evaporation, the air/water interface of the drying droplet moves within the depth of field. The measurements at different heights probe these different droplet regions in more

detail. Whereas placing the focal plane close to the substrate (4 µm) predominantly probes flows occurring in the bulk liquid, a focal plane with a larger distance from the substrate (30 µm) additionally reveals interfacial flows. The data collected at different heights and times for the reference sample clearly reveals that in the bulk of the droplet there is a dominant outward flow towards the droplet edge, which is the cause of the coffee stain effect[12] and is most clearly seen close to the substrate (Supplementary Figure 12, Supplementary Movie 12). In addition, the statistical analysis reveals a backflow along the air/water interface towards the center of the droplet, evidenced by negative radial displacements. This backflow is most pronounced at intermediate and large evaporation times and with a focal plane close to the liquid interface, i.e. at the larger distance of 30 µm from the substrate (Supplementary Figure 13 (d,e)). Supplementary Movie 13 clearly shows the change in the particle movement from an initial outward movement when the focus is in the bulk liquid to an inward movement when the interface comes into focus. This backflow can be attributed to a thermal Marangoni effect, which has been previously calculated[13] and measured[10,11,14,15] by other groups. Both effects lead to an increased particle velocity towards the droplet edge. The capillary flow accelerates particles towards the drying edge, while the Marangoni backflow diminishes at the apex of the droplet where only Brownian motion of the particles is observed (Supplementary Figure 10). Therefore, for the measurements at a height of 30 µm, the decrease in the displacement over time (Supplementary Figure 13,15 (b)) can be explained by a shift in focus from the drop edge towards the apex of the drying droplet in the course of drying, as schematically shown in Supplementary Figure 13,15 (e, top).

Next, we compare the hydrodynamics of the reference sample (Supplementary Figure 12,13; Supplementary Movie 12,13) to the dispersion with PVA-modified particles (Figure 14,15; Supplementary Movie 14,15). Both reference and modified dispersion exhibit a particle population with a positive radial displacement that increases towards the edge of the droplet, which we attribute to the capillary flow of particles in the bulk (Supplementary Movie 12,14). In addition, a second particle population moving towards the apex of the droplet (i.e. with negative radial displacement) can be observed in both dispersions when the focus is closer to the air/water interface (Supplementary Movie 13,15), in particular at later drying stages. We therefore conclude that the water flow within the PVA modified and pure particle dispersion is qualitatively similar. Note that this qualitative interpretation is most clearly seen by the visual impressions of the drying processes shown in Supplementary Movie 12-15.

A quantitative comparison, however, reveals clear differences in the drying behavior of both types of dispersions. For the reference dispersion with pure PS particles, the majority of the particles move radially outwards as a consequence of the capillary flow. This flow increases in the course of the drying and accelerates particles towards the edge where they aggregate to form the coffee stain (Supplementary Figure 12e, Supplementary Movie 12). In the dispersion of PVA-modified particles, a radial outward movement of particles is still present. However, at intermediate and late stages of the drying process, the majority of particles experiences an inward movement and flow towards the droplet center (Supplementary Figure 14-15,d-e). This backflow is evident not only at large distances from the substrate (Supplementary Figure 15), but also close to the substrate (Supplementary Figure 14). We interpret these quantitative differences between reference and PVA-modified particle dispersion as follows. First, the surface-active PVA chains facilitate the interfacial adsorption (see Fig. 3f). Over time, the modified particles are therefore depleted from the bulk and accumulate at the interface, where they are subjected to the thermal Marangoni backflow. This behavior is reflected by an increase in the particle population flowing inward compared to outward, seen in particular at the 30 µm distance to the substrate, where interfacial effects are observed at earlier stages of the drying process (Supplementary Figure 15). Secondly, particles that flow towards the drying edge do not directly aggregate upon contact (as is the case for the reference sample, see Supplementary Movie 6), but retain their mobility, presumably due to the additional steric repulsion by the adsorbed polymer chains (see Supplementary Movie 8, Figure 1d,e). These particles remain in the dispersion close to the droplet edge and subsequently experience a thermal Marangoni backflow, which slowly moves them along the air/water interface back towards the apex of the droplet (see Supplementary Movie 8, Supplementary Movie 14-15). This backward movement is seemingly efficient in redistributing the particles, as evidenced by the large populations with negative displacement, and causes the homogeneous particle distribution after drying.

The different distribution of particles between bulk and interface can also rationalize the quantitative difference in the increase in the average velocity between pure (Supplementary Figure 12b) and modified dispersions (Supplementary Figure 14b). The increase in velocity towards the end of the drying is less steep for the modified sample, because the major fraction of the particles is adsorbed at the air/water interface and the flow rate of the Marangoni backflow is lower compared to the capillary flow. Notably, as more modified particles adsorb at the air/water interface, one would expect a decreased particle adsorption rate. However, simultaneously, the capillary flow (bringing particles towards the air/water interface at the drying front) and Marangoni flow (moving particles along the air/water interface towards the apex) increase with increasing evaporation time.[11] Both effects combined lead to a quasi-linear increase in adsorbed particles over time as observed in Figure 3f.

Interestingly, the thermal Marangoni backflow can be reduced by the presence of small amounts of surfactants.[11] The observation of this thermal Marangoni backflow therefore is an indirect evidence that our particle dispersion is essentially free of unbound PVA, complementing the surface tension measurements of Figure 1b. On the other hand, insufficient cleaning or the addition of free PVA may not only block the air/water interface and prevent particles from adsorbing, but also reduce the thermal Marangoni backflow, leading to a decrease in homogeneity and reoccurrence of the coffee ring (Supplementary Figure 1, 16,18).

Supplementary Discussion 3: Homogeneity of the deposit and effect of particle type and shape

We quantify the homogeneity of the deposits shown in Fig. 2 of the main text by taking SEM images every 80 µm and counting the number of particles on each image using the imageJ tool cell counter. We then calculate the number of particles per unit area, which is plotted in Supplementary Figure 16a,f,k. The microscopy images of the deposit are shown in Supplementary Figure 16b,g,l,p and SEM images at different positions are shown in Supplementary Figure 16c-e,h-j,m-o,q-s.

For spherical PS-PVA particles, we measure a nearly uniform distribution (Supplementary Figure 16a). We hypothesize that since the spherical particles undergo Brownian motion, they are able to maintain a homogeneous assembly at the liquid interface during the drying process. Thermal Marangoni effects provide a flow from the edge towards the center, where diffusion can locally homogenize the interfacial layer. For elliptical PS-PVA particles, we observe a decrease in particles per area towards the center of the deposit. Contrary to spherical PS particles, the ellipsoidal particles aggregate at the liquid interface into chain-like networks due to attractive quadrupole capillary forces.[6–8] We therefore hypothesize that this network structure eventually hinders the Marangoni-driven flow of particles towards the apex of the droplet, and that local equilibration is prevented as the aggregation prevents local diffusion of individual particles. A slightly lower decrease towards the center was observed for SiO_2-graft-PNIPAM particles. SiO_2-PNIPAM-based particle systems are known to aggregate at the liquid interface due to attractive capillary interactions,[4,5] which may also hinder the particle flow towards the apex of the deposit and the local equilibration by diffusion. For pure PS particles we observe a three-dimensional pile-up at the drop edge and essentially no particles within the deposit. The SEM-based counting method is therefore not a suitable method to characterize coffee rings and therefore we do not present a particles per area vs position plot.

Supplementary Discussion 4: Concentration calculations to obtain a particle monolayer:

A suspension droplet on a solid substrate can be described by a spherical cap, where the volume V_L is described by equation (1).

$$V_L = \pi/3 * r_L^3 (1-\cos(\theta))^2 (2+ \cos(\theta)) \quad (1)$$

Where r_L is the radius of the droplet and θ the contact angle between the liquid and the solid substrate. Rearranging equation (1) for r_L leads to:

$$r_L = ((3\ V_L / \pi)/((1-\cos(\theta))^2 (2+ \cos(\theta))))^{1/3} \quad (2)$$

The air/water interface of the droplet M_L, excluding the solid/water interface, is calculated using equation (3).

$$M_L = 2\pi r_L^2 (1- \cos(\theta)) \quad (3)$$

The area occupied by one particle including their polymeric corona at the air/water interface is described by equation (4),

$$A_P = (r_p+t_{corona})^2\ \pi \quad (4)$$

, where r_p is the radius of the particle and t_{corona} the extension of the polymeric corona at the air/water interface. The value for t_{corona} was experimentally determined from optical microscopy images of the interfacial particle assembly shown in Figure 3(ii) using the nearest neighbor distance distribution d_{int} obtained from image analysis software (Figure 3c(ii)), as described in equation (5),

$$t_{corona} = (d_{int} – r_p) / 2 \quad (5)$$

Importantly, since the particles confined at the liquid interface still undergo Brownian motion (Supplementary Figure 10) and therefore are not in direct corona-corona contact, the average nearest neighbor distance would overestimate the size of the corona. For our PS-PVA model system, we therefore chose d_{int} at the onset of the peak in the interparticle distance distribution reflecting the shortest measured distance between two particles, with an estimated value of d_{int} = 1380 nm.

The contact angle of the particle at the liquid interface was simplified to 90°. The number of particles required to fully cover the interface is described by equation (6),

$$N_p = (M_L / A_P)*0.9069 \quad (6)$$

, where A_p describes the interfacial area occupied by one particle including its polymeric corona and the factor 0.9069 describes the maximum packing of spheres in a plane. The total mass of particles in this monolayer m_{ptot} is calculated using equation (7),

$$m_{ptot} = N_p\ (4/3)\ \pi r_p^3\ \rho_P \quad (7)$$

, where ρ_P is the density of the colloidal particles. The concentration c_p in wt-% required to obtain a monolayer on the droplet surface is described in equation (8),

$$c_p = (m_{ptot} / (V_L\ \rho_L))*100 \quad (8)$$

, where ρ_L is the density of the liquid. A comparison to experimental parameters is shown in Supplementary Figure 17. We note that the model neglects the reduction of the liquid interface during the process of drying and the possible compression of the particle corona induced by the same. The compressibility of the particle corona may explain why there is a concentration range, where a homogeneous drying is observed (Supplementary Figure 20b-d).

Supplementary Discussion 5: Exception: Sterically-stabilized particles

In the previous work by Yunker et al.[16] the authors convincingly demonstrated that slightly stretched particles still exhibited a coffee ring and therefore, they attributed the homogeneous drying behavior to the aspect ratio (and with it, the changes in capillary forces) of the ellipsoidal particles. In our work, we do not reproduce this behavior as a homogeneous drying behavior was observed regardless of the particle shape (Figure 2), and also for spherical particles that were embedded in PVA but do not experience any stretching. These contradicting observations are puzzling, and we note that an exact replication and comparison of experiments is difficult as the Yunker publication does not reveal all physicochemical details that may be of importance in the new context of our study. These include about properties of the used PVA (such as molecular weight, which is not of key importance for the stretching process itself), the synthesis protocol or surface chemistry of the commercial microspheres (which often is not available from the data provided by the supplier) and the composition of the dispersion (such as additional stabilizers, possibly added by the supplier) used in their study. However, in the following section we provide a hypothesis that can rationalize the previous results within the framework of the involved physics as demonstrated in our study.

We speculate that the microspheres used by Yunker *et al.*[16] with a diameter of 1.3 µm may have been synthesized by copolymerization with macromonomers,[17] such as mono vinyl-terminated poly(ethylene glycol) (PEG), which is a typical method to obtain larger polymer microspheres with good dispersion stability.[17,18] Such particles can be pictured as “hairy“, with a PS core and dangling PEG chains protruding from its surface.

To investigate how such particles behave in the drying process, we replicate a typical dispersion polymerization of polymer microspheres using a mono vinyl-terminated PEG (2 kDa) as comonomer to introduce steric stabilization, following a literature protocol.[19] The dispersion after synthesis formed a coffee ring upon drying (Supplementary Figure 33b). Noteworthily, the ring pattern appears broader compared to charge-stabilized PS particles, which we attribute to the enhanced steric stabilization that limits aggregation at the drying front, similar to the results we show in Figure 1d. The PEG chain length is too small to provide sufficient interfacial activity to induce a homogeneous drying, corroborating our experiments on the chain length dependence of the polymer additive (Supplementary Figure 32b). Importantly, after the addition of PVA to the particle dispersion and removal of non-adsorbed PVA by centrifugation and redispersion, similar to our protocol in Figure 1, we still observed a coffee ring (Supplementary Figure 33c). These results contrast our findings on charge-stabilized particles reported in Figure 1, but agree with the findings of Yunker *et al*. Since the key difference between the two particle systems are the PEG chains at the surface, we hypothesize that their presence prevents or at least hinders the adsorption of PVA onto the particle surface. We also note that PEG brushes are often used to obtain antifouling surfaces, lending credibility to the decreased adsorption efficiency.[20–22] Upon embedding the particles in a PVA film and stretching into ellipsoids followed by removal of PVA using centrifugation and redispersion in water as followed by Yunker *et al.*,[16] we observed a homogeneous drying behavior of the ellipsoidal particles (Supplementary Figure 33d), again in agreement with the previous results of Yunker *et al*. We hypothesize that this change in drying behavior can result from the change in available surface area, as schematically shown in Supplementary Figure 33a. Upon stretching, new unprotected surface is generated, to which the PVA can adsorb. These additional, longer PVA chains then alter the drying behavior and cause the homogeneous drying pattern, similar to Figure 2. Importantly, as with increasing stretching more free surface is exposed, the amount of adsorbed PVA also correlates with the aspect ratio of the elliptical particles. This change of adsorbed PVA amount may therefore induce the change in drying patterns with the aspect ratio as reported by Yunker *et al.*.[16]

Clearly, this hypothesis remains speculative and necessitates further investigation and quantification, but we believe that the demonstrated effect can reconcile the observations by Yunker *et al.*[16] with our interpretation. In addition, it shows the limits of the described process as a physisorption of PVA onto the particle surface is prevented by the antifouling properties of the PEG hairs and may therefore generally fail with sterically stabilized particle systems.

Supplementary Note 1: Guide for experimentalists

We briefly summarize the criteria that should be considered when designing a particle-polymer-solvent system to achieve a homogeneous drying. Taking together all the experiments and calculations, the criteria required to achieve the described effect can be summarized as follows. We indicate the relevant data supporting the statements in brackets.

1. A surface-active polymer, which can interact with the particle surface and the air/liquid interface, should be chosen (Supplementary Figure 30).
2. The molecular weight of the polymer should be at least 10 kDa (Supplementary Figure 32).
3. The chosen polymer should be dispersible in the same liquid as the particles.
4. The particle system should not already be sterically stabilized with long polymer chains, as they would prevent an adsorption of the surface-active polymer (Supplementary Figure 33).
5. The polymer should adsorb onto the particles, which can be measured by isothermal titration calorimetry (Supplementary Figure 31), by an increase in hydrodynamic diameter measured by DLS (Supplementary Figure 4) or by a change in density and sedimentation coefficient measured by AC (Supplementary Figure 5-7). Importantly, unbound polymer needs to be removed to observe the effect (Figure 1, Supplementary Figure 1, 17, 18). The binding should thus be quasi-irreversible, so that the polymer does not desorb, at least not in the time-frame of the experiment, when unbound polymer is removed, which can be accessed via the binding constant from ITC (Supplementary Figure 31).
6. The polymer should be added to the particle dispersion in excess, as a rule of thumb from our experience approximately 25 times more than required to cover the surface to avoid bridging between particles (Figure 1, Supplementary Figure 18).
7. Non-adsorbed polymer should be removed by cleaning steps until the surface tension of the liquid approaches the values of the pure liquid (Figure 1, Supplementary Figure 1, 34-36). In case of water, the surface tension should be at least > 60 mN/m (Figure 1, Supplementary Figure 1).

Supplementary References

1. Winzen, S. *et al.* Complementary analysis of the hard and soft protein corona: Sample preparation critically effects corona composition. *Nanoscale* **7**, 2992–3001 (2015).
2. Prozeller, D., Morsbach, S. & Landfester, K. Isothermal titration calorimetry as a complementary method for investigating nanoparticle-protein interactions. *Nanoscale* **11**, 19265–19273 (2019).
3. Chiad, K. *et al.* Isothermal titration calorimetry: A powerful technique to quantify interactions in polymer hybrid systems. *Macromolecules* **42**, 7545–7552 (2009).
4. Rauh, A. *et al.* Compression of hard core-soft shell nanoparticles at liquid-liquid interfaces: influence of the shell thickness. *Soft Matter* **13**, 158–169 (2016).
5. Rey, M., Fernandez-Rodriguez, M. A., Karg, M., Isa, L. & Vogel, N. Poly-N-isopropylacrylamide nanogels and microgels at fluid interfaces. *Acc. Chem. Res.* **53**, 414–424 (2020).
6. Loudet, J. C., Alsayed, A. M., Zhang, J. & Yodh, A. G. Capillary interactions between anisotropic colloidal particles. *Phys. Rev. Lett.* **94**, 2–5 (2005).
7. Lim, J. H. *et al.* Heterogeneous Capillary Interactions of Interface-Trapped Ellipsoid Particles Using the Trap-Release Method. *Langmuir* **34**, 384–394 (2018).
8. Botto, L., Lewandowski, E. P., Cavallaro, M. & Stebe, K. J. Capillary interactions between anisotropic particles. *Soft Matter* **8**, 9957–9971 (2012).
9. Marín, Á. G., Gelderblom, H., Lohse, D. & Snoeijer, J. H. Order-to-disorder transition in ring-shaped colloidal stains. *Phys. Rev. Lett.* **107**, 1–4 (2011).
10. Deegan, R. D. *et al.* Contact line deposits in an evaporating drop. *Phys. Rev. E - Stat. Physics, Plasmas, Fluids, Relat. Interdiscip. Top.* **62**, 756–765 (2000).
11. Marin, A., Liepelt, R., Rossi, M. & Kähler, C. J. Surfactant-driven flow transitions in evaporating droplets. *Soft Matter* **12**, 1593–1600 (2016).
12. Deegan, R. D. *et al.* Capillary flow as the cause of ring stains from dried liquid drops. *Nature* **389**, 827–829 (1997).
13. Hu, H. & Larson, R. G. Analysis of the effects of marangoni stresses on the microflow in an evaporating sessile droplet. *Langmuir* **21**, 3972–3980 (2005).
14. Ristenpart, W. D., Kim, P. G., Domingues, C., Wan, J. & Stone, H. A. Influence of substrate conductivity on circulation reversal in evaporating drops. *Phys. Rev. Lett.* **99**, 1–4 (2007).
15. Hu, H. & Larson, R. G. Marangoni effect reverses coffee-ring depositions. *J. Phys. Chem. B* **110**, 7090–7094 (2006).
16. Yunker, P. J., Still, T., Lohr, M. A. & Yodh, A. G. Suppression of the coffee-ring effect by shape-dependent capillary interactions. *Nature* **476**, 308–311 (2011).
17. Kawaguchi, S., & Ito, K. Dispersion polymerization. *Polym. Part.* 299–328 (2005).
18. Shen, R., Akiyama, C., Senyo, T. & Ito, K. Emulsion and dispersion polymerization of styrene in the presence of PEO macromonomers with p-vinylphenylalkyl end groups. *Comptes Rendus Chim.* **6**, 1329–1335 (2003).
19. Harper, G. R. *et al.* Sterie stabilization of microspheres with grafted polyethylene oxide reduces phagocytosis by rat Kupffer cells in vitro. *Biomaterials* **12**, 695–700 (1991).
20. Hamming, L. M., & Messersmith, P. B. Fouling resistant biomimetic poly (ethylene glycol) based grafted polymer coatings. *Mater. Matters* **3**, (2008).
21. Dalsin, J. L., Hu, B. H., Lee, B. P. & Messersmith, P. B. Mussel adhesive protein mimetic polymers for the preparation of nonfouling surfaces. *J. Am. Chem. Soc.* **125**, 4253–4258 (2003).
22. Dalsin, J. L. *et al.* Protein resistance of titanium oxide surfaces modified by biologically inspired mPEG-DOPA. *Langmuir* **21**, 640–646 (2005).
23. Weon, B. M. & Je, J. H. Capillary force repels coffee-ring effect. *Phys. Rev. E - Stat. Nonlinear, Soft Matter Phys.* **82**, 1–4 (2010).
24. Weon, B. M. & Je, J. H. Fingering inside the coffee ring. *Phys. Rev. E - Stat. Nonlinear, Soft Matter Phys.* **87**, 1–6 (2013).
25. Weon, B. M. & Je, J. H. Self-pinning by colloids confined at a contact line. *Phys. Rev. Lett.* **110**, 1–5 (2013).
