# Supplementary material for: Versatile strategy for homogeneous drying patterns of dispersed particles
Source: Nat Commun. 2022 May 23;13:2840. doi: 10.1038/s41467-022-30497-z (PMC9126951; doi:10.1038/s41467-022-30497-z)
Supplement: Supplementary file 3 — Description of Additional Supplementary Files [file 41467_2022_30497_MOESM3_ESM.pdf]

Legends of Supplementary Movies:

Supplementary Movie 1: Accelerated optical microscopy movie of a drying spherical reference PS particle dispersion. The particles accumulate at the drop edge, forming a coffee ring. Drying time: 434 s. Image size: 5.0 x 3.7 mm^2.

Supplementary Movie 2: Accelerated optical microscopy movie of a drying ellipsoidal PS particle dispersion. The ellipsoidal particles still contain adsorbed PVA. The ellipsoidal particles dry out uniformly. Drying time: 421 s. Image size: 5.0 x 3.7 mm^2.

Supplementary Movie 3: Accelerated optical microscopy movie of a drying spherical PS particle dispersion. The particles contain adsorbed PVA. The spherical particles dry out uniformly. Drying time: 417 s. Image size: 5.0 x 3.7 mm^2.

Supplementary Movie 4: Accelerated optical microscopy movie of a drying ellipsoidal PS particle dispersion. Adsorbed PVA was removed. The ellipsoidal particles accumulate at the drop edge, forming a coffee ring. Drying time: 429 s. Image size: 5.0 x 3.7 mm^2.

Supplementary Movie 5: Accelerated optical microscopy movie of a drying spherical PS particle dispersion. Adsorbed PVA was removed. The ellipsoidal particles accumulate at the drop edge, forming a coffee ring. Drying time: 406 s. Image size: 5.0 x 3.7 mm^2.

Supplementary Movie 6: Real-time optical microscopy movie of a drying spherical reference PS particle dispersion at the drop edge. The particles accumulate at the drop edge and form a coffee ring. Image size: 90 x 80 μm^2.

Supplementary Movie 7: Real-time optical microscopy movie of a drying ellipsoidal PS particle dispersion at the drop edge. Adsorbed PVA was removed. The ellipsoidal particles accumulate at the drop edge and form a coffee ring. Image size: 97 x 97 μm^2.

Supplementary Movie 8: Real-time optical microscopy movie of a drying spherical PS particle dispersion at the drop edge. The particles contain adsorbed PVA. The particles adsorb to the air/water interface and assemble in a non-close packed arrangement. Image size: 97x 97 μm^2.

Supplementary Movie 9: Real-time optical microscopy movie of a drying ellipsoidal PS particle dispersion at the drop edge. The ellipsoidal particles contain adsorbed PVA. The particles adsorb to the air/water interface and assemble in a non-close packed side-to-side arrangement. Image size: 97 x 97 μm^2.

Supplementary Movie 10: Real-time optical microscopy movie of a drying ellipsoidal PS particle dispersion at the center of the drop. The ellipsoidal particles contain adsorbed PVA. The ellipsoidal particles aggregate into chain-like aggregates due to strong capillary attraction. Image size: 129 x 97 μm^2.

Supplementary Movie 11: Real-time optical microscopy Movie of a drying spherical PS particle dispersion in the center of the drop. The particles contain adsorbed PVA. The particles adsorb to the air/water interface and undergo Brownian motion, as visualized by the particle tracking method. Image size: 129 x 97 μm^2.

Supplementary Movie 12: Accelerated fluorescent microscopy movie tracking the position of fluorescent tracer particles during drying of a spherical reference PS particle dispersion. The height of the focal plane was set to 4 µm above the substrate. The open circle corresponds to the tracked

position of the particle and the dotted line shows the trajectory of the particles over the last 25 s. Drying time: 260 s. Image size: 891 x 666 μm^2.

Supplementary Movie 13: Accelerated fluorescent microscopy movie tracking the position of fluorescent tracer particles during the drying of a spherical reference PS particle dispersion. The height of the focal plane was set to 30 µm above the substrate. The open circle corresponds to the tracked position of the particle and the dotted line shows the trajectory of the particles over the last 25 s. Drying time: 225 s. Image size: 891 x 666 μm^2.

Supplementary Movie 14: Accelerated fluorescent microscopy movie tracking the position of fluorescent tracer particles during the drying of a spherical PS-PVA particle dispersion. The height of the focal plane was set to 4 µm above the substrate. The open circle corresponds to the tracked position of the particle and the dotted line shows the trajectory of the particles over the last 25 s. Drying time: 279 s. Image size: 891 x 666 μm^2.

Supplementary Movie 15: Accelerated fluorescent microscopy movie tracking the position of fluorescent tracer particles during the drying of a spherical PS-PVA particle dispersion. The height of the focal plane was set to 30 µm above the substrate. The open circle corresponds to the tracked position of the particle and the dotted line shows the trajectory of the particles over the last 25 s. Drying time: 223 s. Image size: 891 x 666 μm^2.
